# WindSeer: real-time volumetric wind prediction over complex terrain aboard a small uncrewed aerial vehicle

**DOI:** 10.1038/s41467-024-47778-4

**Published:** 2024-04-25

**Authors:** Florian Achermann, Thomas Stastny, Bogdan Danciu, Andrey Kolobov, Jen Jen Chung, Roland Siegwart, Nicholas Lawrance

**Affiliations:** 1 Autonomous Systems Lab, ETH Zurich, Leonhardstrasse 21, Zurich, 8092 Zurich, Switzerland; 2 Microsoft Research, One Microsoft Way, Redmond, WA-98052 USA; 3 School of Electrical Engineering and Computer Science, The University of Queensland, Staff House Road, Brisbane, 4072 QLD Australia; 4 CSIRO Robotics, Data61, 1 Technology Court, Brisbane, 4069 QLD Australia

**Keywords:** Fluid dynamics, Aerospace engineering, Computational science

## Abstract

Real-time high-resolution wind predictions are beneficial for various applications including safe crewed and uncrewed aviation. Current weather models require too much compute and lack the necessary predictive capabilities as they are valid only at the scale of multiple kilometers and hours – much lower spatial and temporal resolutions than these applications require. Our work demonstrates the ability to predict low-altitude time-averaged wind fields in real time on limited-compute devices, from only sparse measurement data. We train a deep neural network-based model, WindSeer, using only synthetic data from computational fluid dynamics simulations and show that it can successfully predict real wind fields over terrain with known topography from just a few noisy and spatially clustered wind measurements. WindSeer can generate accurate predictions at different resolutions and domain sizes on previously unseen topography without retraining. We demonstrate that the model successfully predicts historical wind data collected by weather stations and wind measured by drones during flight.

## Abstract (web summary)

Wind has a large impact on the safety and efficiency of small uncrewed aerial vehicles. Here, the authors present a neural network-based method for estimating the wind at meter-scale resolution around complex terrain solely based on terrain knowledge and local onboard wind observations.

## Introduction

Accurate modelling of the wind is crucial for applications such as wind farm layout optimization (WFLO) or safe crewed and uncrewed aviation. The energy generated by wind turbines is proportional to the cubic power of the wind speed; thus, micrositing turbines relies on accurate flow models^1^. Adverse wind poses a challenge for crewed aviation close to the ground at airports with challenging surrounding terrain, such as Madeira International Airport^2^. Finally, winds in mountainous regions can easily exceed 10 ms^−1^, a speed comparable to the normal cruise speed of small uncrewed aerial vehicles (sUAVs)^3,4^, resulting in poor tracking of the planned flight path^5^. If an sUAV knew the wind in advance, it would be able to plan its path so as to avoid areas of unfavorable winds and high turbulence^6^.

Chaotic fluid-dynamic effects due to local steep terrain result in large spatial variations of wind around complex terrain^7^ that require models with high spatial resolution to capture faithfully. Numerical weather prediction (NWP) can accurately model only relatively large-scale wind patterns, with resolutions on the order of kilometers^8^. Computational fluid dynamics (CFD) simulations generate high-resolution wind flows around terrain at smaller scales, on the order of meters or less, but require knowing well-defined boundary conditions reflecting the overall weather situation^9,10^. Both of these classes of simulation-based methods numerically solve the underlying system of partial differential equations (PDEs). Because of this, they are computationally expensive, taking compute times on the order of hours, and do not provide real-time wind field estimates. Onsite wind measurements with a Doppler Lidar^11^ or measurement masts^9,10,12–14^ provide real-time wind information, but at a limited resolution and a high setup cost.

A broad category of methods relevant to our targeted near-terrain wind prediction setting is data assimilation. Data assimilation approaches combine models (such as traditional CFD solutions) with observations to refine prediction based on observed data. Optimization-based methods such as expectation maximization and variational methods use a cost function (generally a metric between the model prediction and observation values) to adapt a model to integrate noisy and sparse observations^15^. Extensive previous work in the NWP domain has focused on temporal (4D-Var) methods that select initial conditions of a model simulation in order to best match temporally and spatially distributed observations^16–18^. However, while assimilation methods can be relatively fast computationally, they require high amounts of input observation data^19^, extensive prior model solutions (such as CFD runs covering the complete area of interest with multiple wind speeds and directions)^20–22^, and/or mesoscale models to act as input boundary conditions^23^. These data and computation requirements are generally unsuitable for online estimation on-board an sUAV due to limited storage and compute that sUAVs tend to carry.

AI-based methods have been used to accelerate the computation of flow fields by assisting or replacing numerical PDE-based solvers in different settings. Examples include modelling fluid flows for visual rendering^24,25^ and replacing CFD simulations in aerodynamic shape optimization^26–30^. However, these models rely on privileged information, such as boundary conditions and consider much simpler geometries compared to the topography of complex terrain. Super-resolution flow analysis closely aligns with our approach, yet previous studies in this field assume complete, uniform coverage of measurements over the entire region^31–34^. They either exclusively investigate two-dimensional flows^31–33^ or require dense low-resolution data^31,33,34^. Various deep neural network (DNN)-based approaches demonstrated weather prediction at a global scale, essentially replacing NWP with much faster compute time in the order of seconds^35–38^. But the resolution of these models, on the order of kilometers, is too low to accurately model the wind around complex terrain.

**Fig. 1:**
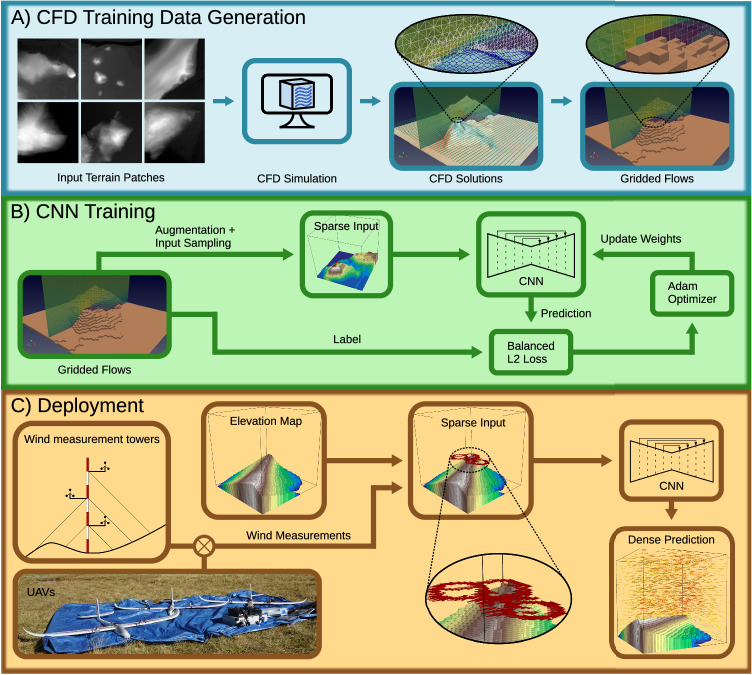
Overview of the wind prediction pipeline. **A** First we generate labelled flows utilizing a CFD simulation. **B** Then WindSeer is trained with measurements along randomly sampled piecewise linear trajectories to predict the dense flow. **C** During deployment the wind estimates from the UAV or wind measurement towers together with the known topography serve as the input to WindSeer.

In this work, we present WindSeer, an approach for predicting the volumetric time-averaged wind and turbulence in real-time at meter-scale based on the topography and sparse, noisy wind observations without needing bulky specialized equipment or assuming access to privileged information. WindSeer’s ability to predict real wind stems from its encoder-decoder convolutional neural network (CNN) architecture trained offline using synthetic flow data generated by computationally expensive steady-state Reynolds-averaged Navier–Stokes (RANS) CFD simulations. The CFD simulations are run offline over real terrain patches that are available from web services^39,40^. The core contribution of our method is in training WindSeer to produce CFD-like predictions from only sparse, in-situ observations – without requiring privileged information such as global boundary conditions. Access to these boundary conditions would be equivalent to measuring the wind along the full boundary of the prediction region, which is simply not available in real-time, nor at the required meter-scale. Once trained, our network-based prediction approach allows for fast, constant-time inference with limited computational and storage resources. An overview of our wind prediction pipeline is presented in Fig. 1 as well as Supplementary Movie 1. We evaluated WindSeer in a series of experiments, whose results show WindSeer’s ability to make accurate dense wind and turbulence predictions based on local noisy wind measurements, across a wide range of spatial resolutions without retraining the model.

## Results

We evaluated WindSeer in a sequence of increasingly challenging experiments to demonstrate its real-time wind prediction capabilities:

1. We demonstrated on held-out CFD-simulated flows that WindSeer is expressive enough to represent the complex flow patterns around real terrain. We analysed several network training approaches on this dataset as it provides dense labels with a wide array of topographies.
2. We demonstrated the ability of WindSeer to predict real wind data using measurements gathered from masts as part of large-scale measurement campaigns over different terrains across Europe^9,10,12–14^, thereby validating both the complete pipeline and our approach of using CFD as a teacher model. These datasets offer good spatial coverage with measurement from different flow regions.
3. On data from several multi-sUAV flights over mountainous terrain we illustrate WindSeer’s ability to predict the time-averaged wind when using noisy onboard wind measurements as input. These input measurements were subject to high noise due to the uncertainty of the estimated sUAV’s pose and errors in the airflow sensing, all of which complicated the prediction problem faced by WindSeer.
4. Finally, we showed WindSeer’s real-time prediction capability on flight-grade hardware.

### WindSeer model

WindSeer is a CNN with four-channel input. The input is composed of a binary mask indicating cells containing input measurements, the terrain model stored as a distance field, and the sparse horizontal wind speed measurements (two channels). Note that vertical wind speed measurements are not an input to the model, since weather stations typically measure only the horizontal wind and the vertical wind is not observable with a standard fixed-wing sUAV sensor set. In Supplementary Note 3 we show empirically that adding vertical wind as an input, if it were available, has a limited impact on prediction quality. The percentage of observed cells in the input data varies across experiments ranging from 3.5 × 10^−6^ % to 0.1%, thus WindSeer always operates on highly sparse observations.

The four-channel output of WindSeer has the same spatial dimension and resolution as the input. The first three channels contain the three-dimensional volumetric time-averaged wind prediction (*W*_*x*_, *W*_*y*_, *W*_*z*_) and the fourth channel contains the turbulence kinetic energy (TKE) prediction — a metric for the strength of the turbulent velocity fluctuations in the wind field that is proportional to the sum of the variances in each dimension. Thus, the prediction contains properties (*W*_*z*_, TKE) that are not available as input measurements.

### Experiment group 1: predicting CFD-simulated flows

We evaluated WindSeer on held-out CFD-simulated flows. The dense label data allowed for evaluation over the full domain, thus evaluating the influence of different measurement locations as well as qualitatively characterizing the prediction quality. We used CFD-simulated flows over previously unobserved terrains and sampled the input measurements and noise from the same distributions as observed during training.

**Fig. 2:**
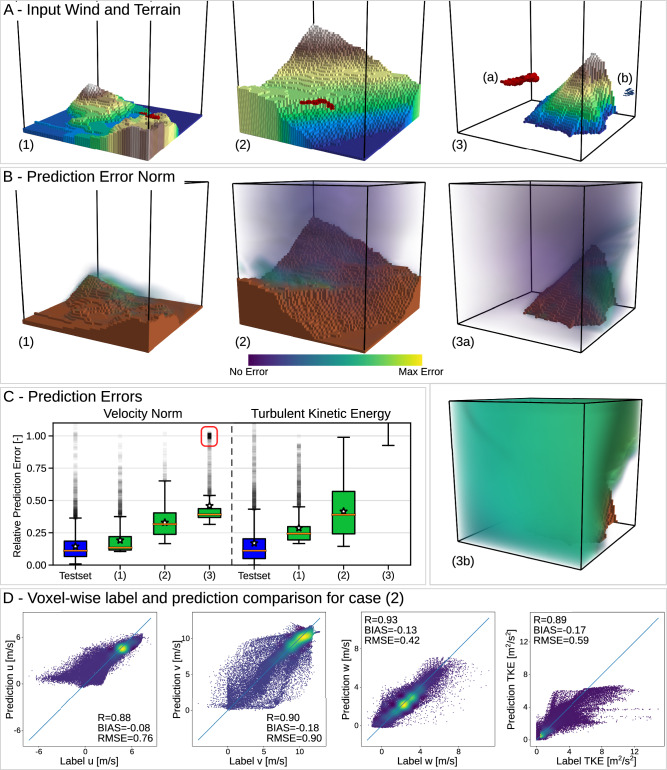
CFD experiment. **A** Terrain and input wind measurements (red arrows) with their respective prediction error. **B** High prediction errors can be observed close to the ground or on the lee side of the terrain. **C** Wind and turbulence prediction performance on the CFD dataset over the full test set containing 4764 samples (blue) and with 2000 random trajectories for the three different terrains shown in (**A**). While most of the terrains result in uni-modal error distributions (1,2), more complex ones can have a second mode for samples from a complex flow region, indicated by the red box in (3). Boxes extend from the first to the third quartiles of data. Median is indicated by a line and mean by a star. Whiskers extend to the extrema data inside 1.5 times the interquartile range beyond the first and third quartiles. Outliers (outside the whiskers) are individually plotted. **D** Density scatter plots (*N* = 64^3^) comparing the label and the predictions for each predicted property using the terrain and input pair presented in (**A**) (2). Source data are provided as a Source Data file.

**Fig. 3:**
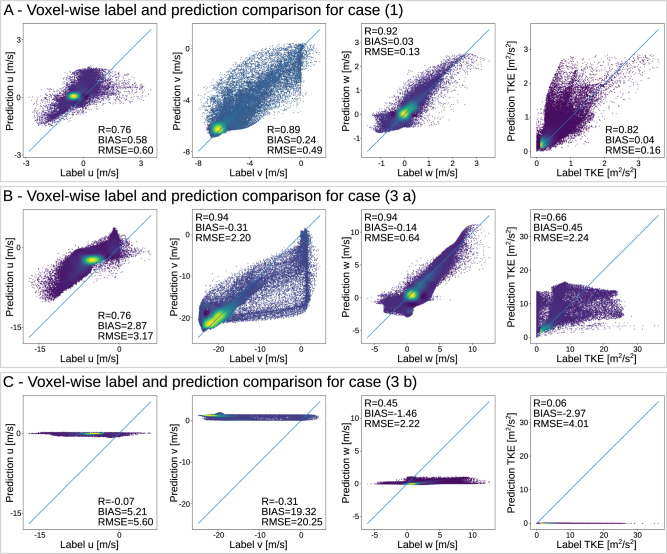
CFD experiment. Density scatter plots (*N* = 64^3^) comparing the label and the predictions for each predicted property using the terrain and input pair presented in Fig. 2A. Panel (**A**) corresponds to case (1), **B** to case (3a), and **C** to case (3b).

Three terrain and input pairs together with the prediction error cloud are shown in Fig. 2A, B. While the highest prediction errors occurred either close to the ground or on the lee side of the terrain, this trend is mitigated by the fact that, due to practical considerations such as payload configuration and safety, the operating altitude for sUAVs is typically over 50 m above ground level^41,42^ and the wind turbine hub heights are typically higher than 80 m above ground^43^. The distribution of the average normalized prediction errors over all non-terrain cells over the full test set is displayed in Fig. 2C in blue. When scoring the network output only above an altitude of 46 m the median relative velocity norm error reduces from 14.5% to 11.5% and the median relative TKE error from 11.2% to 8.3%. The high correlation values (Pearson correlation coefficient) in the voxel-wise comparison of the prediction to the CFD labels shown in Fig. 2D show that WindSeer can qualitatively capture the different flow regimes. The bias (average error) is close to zero for all channels and the root mean squared error (RMSE) is also low compared to the overall magnitude. Together, this underlines WindSeer’s prediction quality. The scatter density plots for the terrains presented in Fig. 2A (1) and (3) are available in Fig. 3.

We evaluated three individual terrains in more detail (Fig. 2A) to assess the sensitivity of the prediction quality to the sampled input data locations. For each terrain we randomly sampled 2000 trajectories and evaluated the prediction error (Fig. 2C) green). No noise or bias was added to the input data in this experiment to focus solely on the impact of the trajectory location. Whenever the input data was sampled in regions where the model could not predict the prevailing flow well, e.g. the lee side of the hill (Fig. 2A) (3b)), WindSeer performed poorly. If such a region is large enough, multi-modal error distributions can be observed as seen in Fig. 2C (3) where prediction failed for input wind samples on the right (lee) side of the hill.

The CFD-simulated flows are computed on a finer grid close to terrain to account for the high spatial variation of the flow in these regions. Interpolating the flow to the coarser fixed-size grid of WindSeer, as visible in Fig. 1A), leads to a loss of information and flow artifacts close to the ground. Such confounding factors make learning these low-altitude flows especially challenging, offering an explanation for the performance difference between predicting the low-altitude and high-altitude winds. However, the experiments with the real wind data show that the wind close to the ground can be accurately modelled if the grid resolution is increased.

### Experiment group 2: evaluation on wind measurement campaign datasets

We evaluated WindSeer on real time-averaged wind data available from three published measurement campaigns. The long measurement periods allow filtering out short-term effects such as wind gusts and reduces the overall measurement noise, enabling an evaluation of WindSeer with clean input wind measurements over larger-scale domains.

**Fig. 4:**
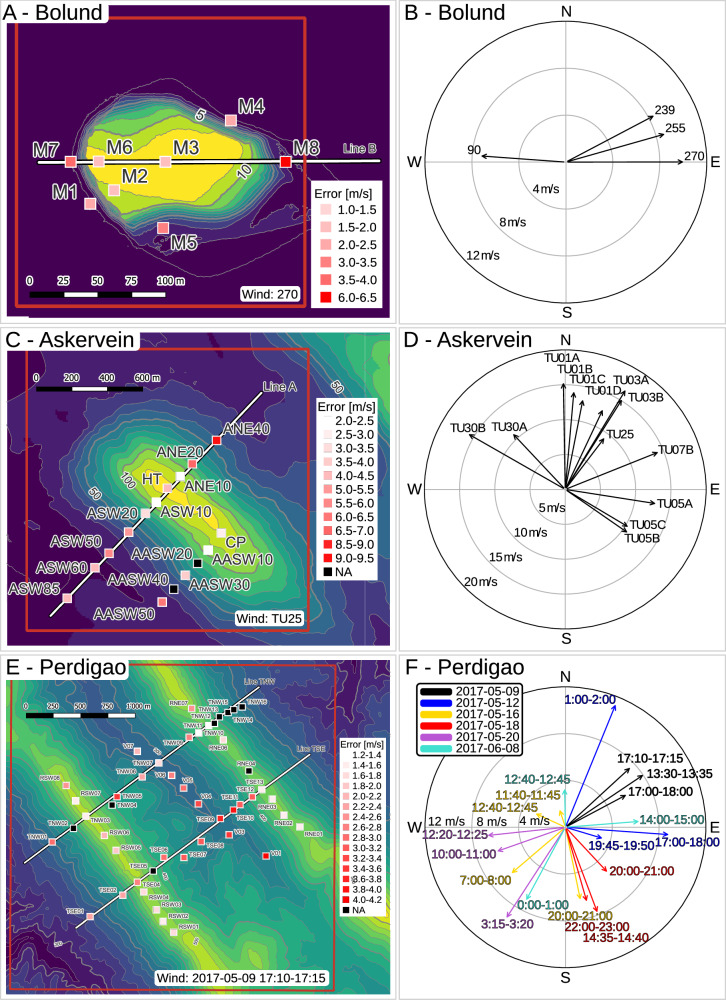
Measurement campaigns experiment. The mast locations and elevation maps for the Bolund (**A**), Askervein (**C**), and Perdigão (**E**) campaigns. The tower positions are colored by the average prediction error when using that specific mast as the input to predict the wind. In the Askervein and Perdigão cases some masts did not provide a valid measurement for that experiment. (**B**, **D**, **F**) show the wind directions for the different experiments for each terrain. Source data are provided as a Source Data file.

The in-situ wind and TKE measurements were collected via wind velocity sensor suites (sonic or cup anemometers) mounted on masts providing data from 2 m to 100 m altitude above ground level^14^. For each terrain, varying wind flow directions and magnitudes are available from different measurement periods (Fig. 4). The terrain in these campaigns varies in complexity and size – from the 11 m high Bolund hill (Fig. 4A)^9,10^ to the gently-sloped 116 m high Askervein hill (Fig. 4C)^12,13^. Both Bolund and Askervein have limited vegetation while the Perdigão region in Portugal represents the most complex test case with two lightly-forested parallel ridges roughly 300 m high (Fig. 4E).

The varying geometric extents of the sites together with the low altitude wind measurements required a larger grid size (384 × 384 × 192 instead of 64^3^ cells) paired with higher resolution to obtain meaningful predictions. Accordingly, the grid resolutions were increased 2 × , 4 × , and 30 × for the Perdigão, Askervein, and Bolund terrains, respectively. These changes in the prediction grid were enabled by the fully convolutional architecture of WindSeer and the distance field representation of the terrain that indirectly provides the cell size and resulted in around 100 × sparser input data compared to the training density.

We compared WindSeer against an averaging baseline (AVG) that assumes the wind and TKE are constant and predicts the average of all measurements over the full domain. This is a widely accepted assumption for sUAV flights^5,44,45^, moreover, the state of the art in planning large-scale missions relies on NWP forecasts that remain constant at the spatial resolution of the mission^46^. Each method predicted the wind based on the measurements from a single mast, yielding an ensemble of predictions for each wind case, while measurements from the remaining masts were used to validate the predictions.

For each wind case we averaged the metrics over the ensemble of predictions and report the absolute errors of the wind magnitude, vertical wind, and TKE in Table 1. In most cases WindSeer outperformed the averaging baseline (AVG). In the other cases, the wind direction usually aligned with the ridge/terrain, causing only small variations across the measurements of the different masts. The average prediction errors of WindSeer are 17%, 43%, and 39% lower than the baseline for the velocity magnitude, vertical wind and TKE respectively.

**Table 1:** Real world wind results

		S [*m*/*s*]			W [*m*/*s*]			TKE [*m*^2^/*s*^2^]		
		AVG	WindSeer		AVG	WindSeer		AVG	WindSeer	
Terrain	Case	*ϵ*	*ϵ*	*ρ*	*ϵ*	*ϵ*	*ρ*	*ϵ*	*ϵ*	*ρ*
Bolund	90	1.80	**1.58**	0.72	0.85	**0.58**	0.50	1.91	**1.33**	0.58
	239	2.80	**2.50**	0.68	0.66	**0.34**	0.76	2.67	**1.68**	0.86
	255	3.24	**2.47**	0.82	0.85	**0.44**	0.72	3.43	**2.12**	0.82
	270	3.77	**2.79**	0.85	0.95	**0.51**	0.78	5.14	**3.43**	0.73
Askervein	TU25	2.58	**2.39**	0.65	1.10	**0.37**	0.90	0.61	**0.41**	0.89
	TU30A	1.14	**0.98**	0.62	0.41	**0.26**	0.58	1.38	**0.72**	0.42
	TU30B	1.80	**1.46**	0.73	0.51	**0.41**	0.64	2.82	**1.40**	0.23
	TU01A	3.26	**2.83**	0.77	1.41	**0.52**	0.91	1.89	**1.06**	0.85
	TU01B	3.24	**2.74**	0.79	1.37	**0.48**	0.92	1.64	**0.98**	0.87
	TU01C	3.55	**3.08**	0.78	1.23	**0.45**	0.92	1.17	**0.72**	0.90
	TU01D	4.21	**3.71**	0.79	1.26	**0.47**	0.93	1.62	**1.17**	0.93
	TU03A	5.29	**4.70**	0.78	1.74	**0.64**	0.93	2.04	**1.31**	0.98
	TU03B	4.90	**4.41**	0.77	1.54	**0.54**	0.92	1.82	**1.21**	0.90
	TU05A	1.91	**1.89**	0.62	0.76	**0.31**	0.89	1.73	**0.99**	0.40
	TU05B	1.18	**1.00**	0.79	0.31	**0.26**	0.48	1.40	**0.63**	0.04
	TU05C	0.93	**0.93**	0.66	0.34	**0.25**	0.58	1.09	**0.43**	0.14
	TU07B	3.42	**3.27**	0.70	1.59	**0.49**	0.90	2.44	**1.85**	0.40
Perdigão 2017-05-09	13:30–13:35	2.91	**2.27**	0.82	0.85	**0.57**	0.53	-	-	-
	17:10:17:15	4.41	**3.33**	0.48	1.22	**0.88**	0.35	-	-	-
	17:00–18:00	3.06	**2.37**	0.77	0.80	**0.56**	0.50	-	-	-
Perdigão 2017-05-12	01:00–02:00	2.82	**2.31**	0.76	0.52	**0.42**	0.45	-	-	-
	17:00–18:00	2.76	**2.23**	0.81	0.70	**0.57**	0.57	-	-	-
	19:45–19:50	1.15	**0.90**	0.71	0.21	**0.16**	0.57	-	-	-
Perdigão 2017-05-16	07:00–08:00	1.58	**1.16**	0.65	0.27	**0.27**	0.67	-	-	-
	11:40–11:45	0.85	**0.77**	0.51	0.33	**0.27**	0.22	-	-	-
	12:40–12:45	0.86	**0.75**	0.48	0.29	**0.22**	0.30	-	-	-
	20:00–21:00	1.35	**0.97**	0.68	**0.22**	0.31	0.21	-	-	-
Perdigão 2017-05-18	14:35–14:40	2.14	**1.82**	0.70	0.41	**0.36**	0.33	-	-	-
	20:00–21:00	1.54	**1.08**	0.84	**0.17**	0.19	0.12	-	-	-
	22:00–23:00	1.52	**1.13**	0.74	0.17	**0.17**	0.42	-	-	-
Perdigão 2017-05-20	03:15–03:20	3.59	**2.63**	0.61	**0.41**	0.55	0.30	-	-	-
	10:00–11:00	2.20	**1.84**	0.71	0.51	**0.42**	0.46	-	-	-
	12:20–12:25	1.93	**1.71**	0.66	0.58	**0.43**	0.45	-	-	-
Perdigão 2017-06-08	00:00–01:00	2.68	**1.87**	0.69	**0.35**	0.38	0.44	-	-	-
	12:40–12:45	1.20	**0.90**	0.77	0.31	**0.22**	0.46	-	-	-
	14:00–15:00	2.49	**1.77**	0.82	0.74	**0.42**	0.58	-	-	-
All campaigns		2.50	**2.07**	0.71	0.72	**0.41**	0.59	2.05	**1.26**	0.64
Chasseral	Flight 1	**0.61**	0.85	0.33	0.54	**0.38**	0.95	-	-	-
	Flight 2	**0.57**	0.77	0.48	0.50	**0.32**	0.80	-	-	-
	Flight 3	**0.53**	0.71	0.37	0.49	**0.30**	0.84	-	-	-
Oberalp	Flight 1	**0.56**	0.82	−0.46	0.54	**0.33**	0.78	-	-	-
Gotthard	Flight 1	**0.99**	1.13	0.33	0.21	**0.18**	0.88	-	-	-
All flights		**0.65**	0.86	0.21	0.46	**0.30**	0.85	-	-	-

Absolute prediction errors (*ϵ*) and correlation between the measurements and predictions (*ρ*) for the velocity magnitude (S), vertical wind component (W), and turbulence kinetic energy (TKE) on the measurement campaign datasets and flight experiments of WindSeer compared to the averaging baseline (AVG). The best performing model for each case is highlighted bold. Note that the AVG baseline does not offer correlations as it produces a constant signal.

To assess whether WindSeer can predict flow trends well, we also report the correlation between the wind predictions and measurements in Table 1. The correlation for the AVG baseline is undefined, due to the constant, location-invariant wind prediction. Averaged over all cases, WindSeer yielded strong positive correlations for all metrics suggesting that it was able to predict the observed trends well, such as up- and downdrafts, high wind and turbulence, thus providing a valuable contribution to planning safer and more efficient sUAV trajectories or WFLO.

**Fig. 5:**
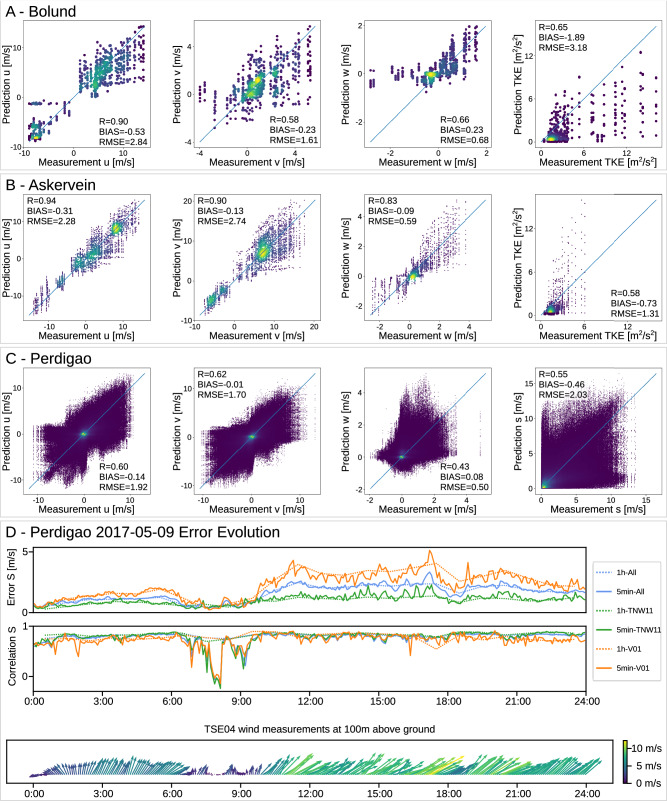
Measurement campaign results. Measured wind compared to the predictions aggregated over all predictions for the Bolund (**A**, 32 predictions [4 experiments, 8 masts]), Askervein (**B**, 182 predictions [13 experiments, 14 masts]), and Perdigão (**C**, 9120 predictions [240 experiments, 38 masts]) campaigns. In (**D**) the evolution of the prediction error and correlation of the wind norm S for WindSeer (WS) using the 5 min and 1 h averaged data together with the measurements from the TSE04 tower as a reference are shown. We show the results aggregated over all the 38 predictions using the different masts as input and the scores using only the TNW11 and V01 tower data for the prediction. Source data are provided as a Source Data file.

In Fig. 5A–C we compare the measurements to the WindSeer predictions. Analogous to the previous experiment we use the wind measurements from one mast as input resulting in 32 predictions for the Bolund [8 masts and 4 wind cases], 182 predictions for the Askervein [14 masts and 13 wind cases], and 9120 predictions for the Perdigão campaigns [38 masts and 240 wind cases (one hour averaged data for ten different days [2017-05-09, 2017-05-11, 2017-05-12, 2017-05-16, 2017-05-18, 2017-05-20, 2017-05-26, 2017-06-02, 2017-06-03, 2017-06-08)]. Overall, the low bias and RMSE together with the high correlation demonstrate that WindSeer handles different wind conditions well. The best predictions are obtained for the simpler Askervein terrain and the errors grow with increasing terrain complexity. In general the model under-predicts the downdrafts in the wind fields for the Bolund and Perdigao cases (Fig. 5A, C) but the Askervein experiment (Fig. 5B) shows that WindSeer is capable of representing strong downdrafts.

For the Bolund hill a study of different CFD simulation was conducted and the speed up errors for one wind direction (239^°^) are reported^10^. The average speedup prediction error for WindSeer is 20.3% while the best performing RANS-CFD models achieve an error of 15% but with runtimes in the order of hours. The models with a runtime of less than 15 min have errors of 26.5% to 32.4%, comparable to our averaging baseline with error 33.5%.

The RANS CFD simulations compute the time-averaged solution of the Navier-Stokes equation, thus WindSeer is trained to operate on these static flows. However, in the real world, wind changes constantly. The Perdigão campaign provides measurements as 5 min averages allowing us to compare the performance of WindSeer operating with high- and low-frequency data as shown in Fig. 5D. Averaged over a day, the performance is consistent across the two time windows with slightly better results using the hourly averages. One exception is the large difference in the correlation scores observed between 06:00 and 10:00, a time period characterized by low wind magnitudes and rapidly changing wind direction as evident from the TSE04 tower measurements. These dynamic conditions do not match the RANS CFD simulation offering an explanation for the poorer prediction performance on the 5-minute averaged data compared to the hourly averages that filter out these unsteady flow features.

In Fig. 4 the masts are colored by the averaged wind magnitude error of the WindSeer prediction when using the measurements from that respective mast as inputs. Lighter colors indicate input measurement mast locations that yielded more accurate predictions. In Fig. 5D we show the prediction errors and correlation using the TNW11 and V01 tower data in addition to the averaged errors over all tower predictions. Consistent with the findings from our previous experiments, using measurements from the hill top or the upwind side generally resulted in lower-error predictions than measurements from the lee side, nevertheless, the correlation is consistently high.

**Fig. 6:**
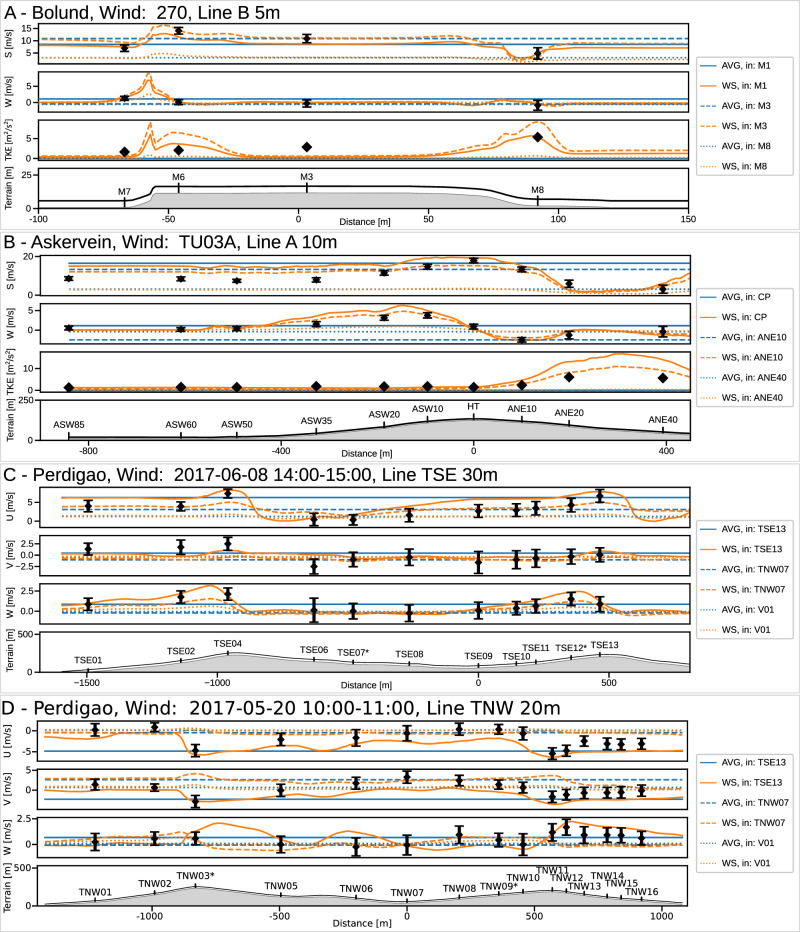
Measurement campaign results. Predictions and measurements along characteristic lines with a constant height for a Bolund hill case (**A**), Askervein hill case (**B**), and two Perdigão cases (**C**, **D**) with the baseline averaging method (AVG) and WindSeer (WS). Three predictions using different input masts are shown for each model and experiment. The asterisk * indicates that no measurement was available for that respective mast at the queried height and the closest one was picked. The uncertainty of the measurements is displayed by the standard deviation of the raw high-rate data. In (**D**) the measurements indicate a rotor between the two ridges, a flow pattern not present in the training data. Thus, WindSeer struggles to accurately represent the flow for the towers TNW06-TNW10.

In each campaign the masts were arranged along multiple straight lines enabling us to qualitatively assess whether the models could capture expected flow trends along these lines. We selected cases where the wind and the line direction are parallel, as the measurements show higher variation in these scenarios, and present the WindSeer and baseline predictions in Fig. 6. Refer to Fig. 4 for the wind direction and the mast locations. For each method and case we show three predictions using the measurements from different masts as the input. The error bars for wind speed measurements report the 1*σ* uncertainty.

WindSeer successfully predicted the speed changes and up-/downdrafts unless the measurement tower was located on the lee side of a hill, e.g. Bolund: M8, Askervein: ANE40, Perdigão: V01/TNW07. Wherever TKE measurements were available, WindSeer predicted TKE trends well. WindSeer struggles to predict flow patterns not observed during training, such as a lee side rotor which occurs when flow detaches on the downwind side and causes a recirculating pattern (Supplementary Note 1). Such a case is shown in Fig. 6D.

### Experiment group 3: Predicting the wind along sUAV trajectories

We evaluated WindSeer on noisy, local wind measurements collected by multiple fixed-wing sUAVs. This mirrors the targeted use case of WindSeer and challenges it with high input noise levels due to real sensor noise and short term wind effects such as gusts or turbulence.

We flew multiple fixed-wing sUAVs simultaneously in the Swiss Jura (Chasseral) and the Swiss Alps (Oberalppass and Gotthardpass). The flight plans for each sUAV consisted of multiple circular loiter patterns. We generated the sparse input from the point-cloud of measurements to WindSeer by binning the observations along the flight path from one sUAV into the discretized prediction grid and averaging all measurements falling in a single cell. We used the native training grid size and resolution with the grid center at the first observed sUAV position estimate.

We first evaluated WindSeer on time-averaged data, similar to the previous experiment group with the static masts, to reduce the noise and sensitivity to sensor calibration on the input measurements. We generated this data by averaging the measurements over one loiter pattern to a single wind measurement. The observations from the different sUAVs and multiple loiters enabled us to compute the average prediction error and the correlation for each flight experiment. We present the error metrics for all flights in Table 1. For the wind magnitude, the baseline outperforms WindSeer which also only yields a slightly positive correlation averaged over all flights. Nevertheless, the high correlations and significantly lower errors for the vertical wind compared to the baseline indicate that WindSeer can better predict the locations of dangerous downdrafts, as well as favourable updraft regions, based solely on wind measurements taken from one sUAV.

**Fig. 7:**
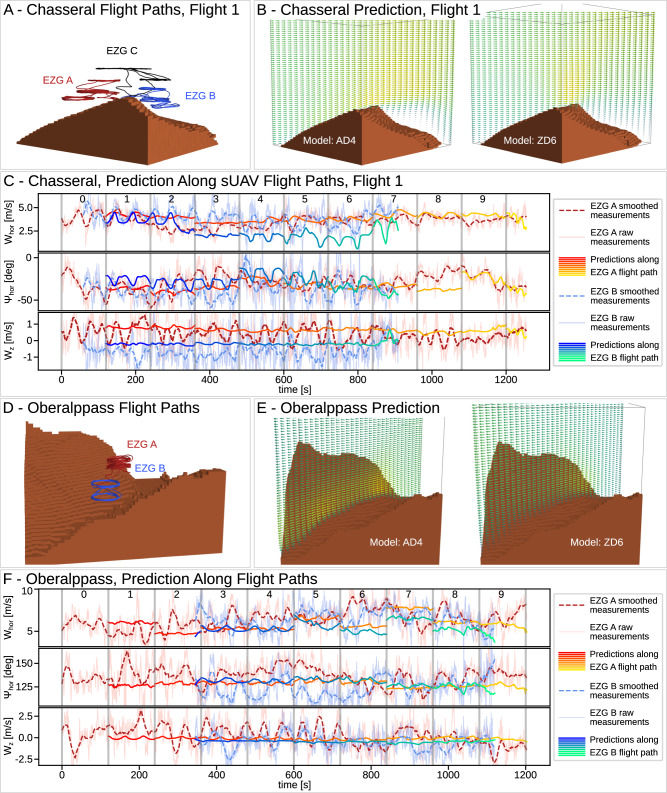
sUAV flight experiment. Prediction results and flight paths for two flight tests: Chasseral (**A**–**C**) and Oberalppass (**D**–**F**). The predictions along a slice are shown for the AD4 and ZD6 models (**B**, **E**). **C**, **F** Show the sliding window predictions of the ZD6 WindSeer variant along the flight paths using the data from EZG A as input. Every 120 s a prediction is made using the wind data from the previous window.

**Fig. 8:**
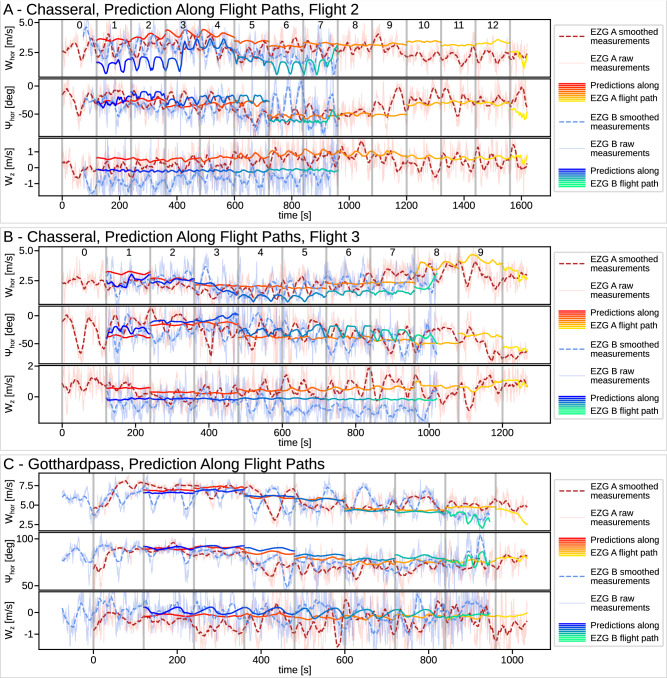
sUAV sliding window predictions. Additional sliding window predictions of the ZD6 WindSeer variant along the flight paths for the second (**A**) and third (**B**) Chasseral flights and the Gotthardpass flight (**C**) using the data from EZG A as input. Every 120 s a prediction is made using the wind data from the previous window.

We further evaluated WindSeer in a sequential time-windowed manner using the wind data from a 120 s window to predict the wind along the flight trajectory for the next window. The corresponding predictions for the test flight are displayed in Figs. 7, 8. Note that the prediction between successive windows can be discontinuous due to the different measurements used by WindSeer. The model was able to accurately predict the magnitude difference in the vertical wind between the two sUAVs for all Chasseral flights. It slightly under-predicted the downwind on the lee side for the validation sUAV, which can be explained by the generally worse performance of the models on the lee-side wind predictions, as shown in the previous experiments.

Although the measurements were averaged over time the noise due to wind gusts and measurement errors was comparable to the variation of the measured wind magnitude as outlined in Supplementary Note 4. Thus, the averaging already offered a good baseline prediction of the wind magnitude. As the wind on the vertical axis varied much more between the different sUAVs and throughout a flight, WindSeer could predict these variations and outperform the baseline.

The Oberalppass and Gotthardpass are especially challenging prediction terrains, as they exhibit large altitude changes (1500 m) due to valleys and peaks within 4 km of our flight locations. High surrounding peaks can cause high gust levels, explaining the high variation in the measured wind. Furthermore, terrain outside the prediction area can significantly influence the wind features observed in the valley. In contrast, our CFD simulation setup for generating the WindSeer training data was limited to well-defined inflow conditions and a domain size of 1.5 km × 1.5 km, which did not allow simulating the flow over multiple large scale mountains or ridges. Thus, during training WindSeer did not observe such complex wind flows, explaining the performance difference between the Chasseral and Oberalppass/Gotthardpass flights.

### WindSeer inference time

We evaluated prediction times of WindSeer on an NVIDIA Jetson Orin AGX, a light-weight and low-power single-board computer, to show real-time performance on sUAV flight-grade hardware. The average inference times over 100 runs on a 64^3^ and 384 × 384 × 192 prediction domain were 0.021 ± 0.002 s and 3.577 ± 0.015 s respectively. Mixed-precision inference reduced the inference times to 0.021 ± 0.005 s and 1.700 ± 0.005 s. These inference times show that WindSeer is capable of low-latency wind predictions over large domains with limited compute to quickly recalculate predictions in response to new measurements.

## Discussion

In this work, we have proposed an approach to train a CNN, WindSeer, for predicting low-altitude time-averaged wind and TKE around complex terrain in real-time based on sparse and noisy wind measurements and known topography. We trained WindSeer solely on simulated steady-state RANS CFD flows over terrain patches from Switzerland and evaluated it on held-back CFD data and real wind measurements. In the first experiment on previously unobserved CFD solutions we demonstrated that WindSeer is capable of replicating the dense flows based on sparse and noisy observations with high accuracy (median relative error below 10%).

In the next experiments we demonstrated zero-shot sim-to-real transfer by evaluating WindSeer on real wind measurements without retraining. On the historic measurement campaign datasets we showed that WindSeer was able to reconstruct real wind flows of different scales, at up to 30 times higher resolution than the training data. This corresponds to larger prediction domains containing up to 108 times more cells than those used for network training and to much sparser input data compared to training. The distance field representation enabled this multi-resolution property of WindSeer as it provided a sense of scale to the model. In summary, this allows for customized prediction grids to suit various scenarios based on the necessary resolution and extent.

Finally, the performance of WindSeer on the flight data is comparable to the average baseline assumption, with less accurate predictions of the horizontal wind but superior performance when predicting the vertical wind, which is the key factor when assessing the safety and efficiency of flight plans. The flight data exhibits much higher measurement noise due to the noise of the low-cost sensors used to estimate the sUAV pose and wind. Therefore, until better wind sensing is available on sUAVs, we envision the onboard deployment of WindSeer by using the weather data from nearby weather stations.

Previous work has shown the ability of DNNs to predict fluid flows for well-defined geometries paired with well-known inflow conditions^26–28,47,48^. We demonstrated the capability of DNNs to work with sparse and noisy input data on realistic complex terrain. This enables real-time wind prediction using data that is feasible to obtain aboard an sUAV. The sparsity of the input data (0.19% down to 3.5 × 10^−6^ %) exceeds previous research of sparse-to-dense DNNs that usually assume denser data around 0.75%^49,50^, 0.2%^51^, or 6.5 × 10^−3^%^52^. In these previous examples the sparse input data was distributed over the whole prediction domain while in our case we showed WindSeer still performs well even if the samples are located within a spatially constrained sub-region.

### Limitations and future work

#### Training domain

In our data generation pipeline we restricted the CFD simulation domain to 1.5 km × 1.5 km based on the initial assumption that the large scale NWP would be used in the network input (Supplementary Note 2). This domain size restricted the terrain to mostly contain one single major geographical feature such as a mountain or a ridge. Therefore the current CFD training data does not contain samples that include wind phenomena such as lee-side rotors, which arise in the presence of multiple mountains/ridges. A larger simulation domain in the order of 10 km × 10 km could allow a better representation of such complex flows and possibly increase the wind prediction performance for complex terrains inside mountain ranges. A biased sampling strategy when composing the input could also help to solve the sample imbalance problem by exposing the network to more examples from the lee-side flow regime during training.

#### Temporal wind variation

Changing the CFD simulation from a time-averaged RANS solution to a time-varying model such as large eddy simulation (LES) or a mesoscale weather prediction, such as the WRF model^53^, could have multiple advantages. First,  the DNN could be trained using the time-varying wind data to construct the input but still predict the time-averaged solution. This could result in the model learning wind gust characteristics and thus increase robustness to noisy wind estimates from the sUAV. Second, a model could be trained to predict the time-varying flow representing wind gusts and short term weather evolution in the predicted wind. However, whether the information from the noisy measurements is sufficient to uniquely determine the flow state still needs to be carefully analyzed. Depending on the sparsity of the data there are likely to be multiple possible flow solutions matching the observations. Further, time varying methods like LES require significantly more computational resources than RANS solvers, further increasing the cost of generating training data^54^.

#### Fluid flow assumptions

Currently the CFD simulations used to train WindSeer model the air as an incompressible fluid with uniform temperature. By including temperature differences in the compressible fluid and terrain the CFD simulation could model complex flow phenomena such as thermals^55^, updrafts caused by temperature variations on the ground, or mountain waves^56,57^, which are large scale oscillations of the wind direction and magnitude behind large ridges. However, simulating these phenomena would require far more input data and ultimately we would still need to verify whether these simulations provide realistic flows that reflect the true airflow characteristics.

#### Wind estimation

Our current wind sensing setup onboard the sUAV is prone to calibration errors and noise resulting in relatively large wind estimation errors. Alternative sensors, such as a five-hole probe^58^ paired with an improved calibration procedure or better sensor placement could improve the wind estimates.

## Methods

### Overview

We developed a pipeline (Fig. 1) to train and deploy a CNN, WindSeer, that predicts the dense time-averaged wind and turbulence around complex terrain. The network training consists of two steps: First we generated a dataset of dense flows over terrain patches from Switzerland using a RANS CFD solver (Fig. 1A). We then trained WindSeer using the label flows to simulate local wind measurements along randomly generated piecewise-linear trajectories, robustifying the predictions by adding noise to the measurements along the trajectories (Fig. 1B). The trained WindSeer was evaluated on (i) held back CFD-simulated flows on previously unobserved terrains, (ii) real wind data gathered in measurement campaigns^9,10,12–14^, and (iii) real wind data measured by multiple sUAVs around mountainous terrain.

### CFD wind data

We generated flow data over real terrain patches with a pipeline based on the open source solver OpenFOAM^59,60^ with the steady-state RANS model and the popular *k* − *ϵ* two-equation turbulence closure^61^. The automated pipeline ingests terrain patches and outputs the time-averaged flow solutions for multiple wind speeds^62^. We extracted 563 terrain patches each with an extent of 1.5 km × 1.5 km from the GeoVite service (https://geovite.ethz.ch/), which provides access to the swissALTI3D digital elevation map (DEM) for Swiss researchers, with a lateral resolution of 0.5 m, recently also available on ArcGIS (https://elevation.arcgis.com/arcgis/rest/services/WorldElevation/Terrain/ImageServer). The terrain patches exhibit at least one side with near-constant elevation allowing us to simulate a formed boundary layer flow (logarithmic profile) entering into the domain from that face. Some terrains allowed for multiple flow directions leading to 866 terrain/flow direction pairs. The vertical extent of the simulation domain was three times the height difference of the terrain with a lower bound of 1100 m minimizing the boundary effects on the flow. Each case was simulated with up to 15 different wind speeds if the automatic meshing succeeded, resulting in 7361 executed CFD runs. We initialized the subsequent simulation for the higher wind speed cases with the previous solution to speed up computation. Only solutions that met a required optimization tolerance were accepted as fully converged solutions, which was the case in 92.9% of the runs. We enhanced our dataset with one zero-velocity flow for each terrain that had at least one converged CFD simulation, resulting in a total of 7285 flows.

The CFD solutions are computed on an automatically generated irregular mesh with OpenFOAM’s SnappyHexMesh utility. We resampled each case up to a height of 1100 m to a regular 91 × 91 × 96 grid resulting in a resolution of 16.5 m horizontally and 11.5 m vertically (Fig. 1A).

### Data augmentation

Generating CFD flows is a computationally and labor-intensive task. For reference, our 7361 CFD runs required 9168 h CPU compute time (782 h creating the meshes and 8386 h solving the flow, average compute time: 1.25 h). Unfortunately, deep networks are notoriously data-hungry and, for a complex modeling problem such as wind prediction, would typically require orders of magnitude more training data to achieve good performance. In computer vision, image augmentation methods are widely used when training deep CNNs^63^. These methods aim to improve the quality and size of the datasets when only limited data is available to prevent the networks from over-fitting. In this work, we showed, for the first time, that geometric transformations can be applied to CFD flows to augment the WindSeer training data.

We randomize the locations of terrain features and flow directions by generating 64^3^ subdomains sampled from each full 91 × 91 × 96 grid. The subdomains are constructed by sampling from a range of rotations and origin translation offsets inside the full domain. In a first step the horizontal shift and a rotation around the *z*-axis are sampled from bounded uniform distributions to ensure that the shifted and rotated 64^2^ subdomain is fully contained within the full 91^2^ domain. Then in a second step the vertical shift is sampled from a triangle distribution with lower limit and mode of 0 and an upper limit of 32. Smaller vertical offsets are favoured to focus on the complex flow regions closer to the terrain. The flow data is linearly interpolated to the coordinates of the subdomain grid, which is the same spatial resolution as the full grid.

### Input and label composition

The input to WindSeer consists of four volumetric channels, one of which corresponds to the terrain encoding *T* created as a Euclidean distance transform with zeros inside the terrain. That representation propagates the terrain information over the full domain and even allows us to include terrain features outside of the domain if they are accounted for in the distance field calculation. The remaining three channels include the sparse and noisy horizontal wind measurements (*U*_*x*,*in*_, *U*_*y*,*in*_) and a binary mask *B* indicating cells containing measurements. Previous work has shown the value of providing binary input masks to CNNs handling sparse input data^64,65^.

Measurements from weather stations or realistic flight scenarios only cover a small percentage of the prediction volume along a connected path, e.g. a 30 s flight segment with our sUAV covers approximately 20 cells at the default grid resolution. Consequently, for a practical onboard wind prediction scenario, we expect the available input wind data to be very sparse and thus construct our network input to reflect this sparsity. We create the input based on the augmented dense flow by creating a mask and then selecting the measurements based on the mask. We emulate the characteristics of an sUAV flight path by filling the mask along sequential randomly-selected piecewise linear segments with a length of 3 to 500 cells.

Noise is added to the sampled wind data in order to account for fluctuations in the wind and sensor errors that are not captured by the RANS CFD simulations. Two types of disturbance are added, white Gaussian noise (sampled i.i.d. at each measurement from ${{{{{{{\mathcal{N}}}}}}}}\left(0,{\sigma }_{g}^{2}\right)$) and measurement bias (sampled from ${{{{{{{\mathcal{U}}}}}}}}\left(-0.1,0.1\right)$ and applied to all measurements). The first has the purpose of simulating noise due to sensor measurements^66^, while the latter simulates the effects of sensor miscalibration. The standard deviation for the Gaussian noise *σ*_*g*_ itself is drawn from a uniform distribution: ${\sigma }_{g} \sim {{{{{{{\mathcal{U}}}}}}}}\left(0,0.1\right)$, simulating different noise levels. All the noise values are scaled with the mean wind velocity for each sample in the training set to have coherent noise levels from low to high velocity samples. Note that noise is only added to the training inputs and not to the CFD ground truth labels used to compute the network training losses.

The sparse input implies that for most cells in the input wind velocity channels (*U*_*x*,*in*_, *U*_*y*,*in*_) the values are undefined since they do not contain a measurement. We test and evaluate two approaches to filling the missing information. The first naïve approach simply places zeros in all voxels without a measurement. This results in large gradients of the input for high magnitude wind. The second approach uses the per-channel-average of all measurements as the fill value resulting in a smoother input and propagating the information over the whole domain.

The labels are constructed by stacking the four volumetric channels corresponding to the three-dimensional predicted velocity (*U*_*x*,*out*_, *U*_*y*,*out*_, *U*_*z*,*out*_) as well as the TKE at each cell from the CFD ground truth flows.

### Model training

The wind prediction model is an encoder-decoder CNN with skip connections based on the U-Net architecture^67^. The WindSeer encoder is composed of single 3D convolutions with kernel size 3 and reflection padding to preserve the size. Using skip connections, the information at each depth is relayed to the decoder before utilizing a max-pooling layer with kernel size of 2 to down-sample the feature map. The original domain size is restored by pairing the information from the skip connection with a nearest-neighbor up-sampled feature map followed by two 3D convolutions with kernel size 4. This decoder structure removes checkerboard artifacts sometimes experienced when using an encoder-decoder CNN^68^. Each convolution, except the final one, is followed by the nonlinear ReLu layer with negative slope 0.1^69^.

A majority of the cells in the wind speed input channels contain no measurements. Our first approach was to set the values of all these cells to zero. However, this resulted in a network overfitting to the number of observed cells and did not generalize to larger domains and different resolutions as demonstrated in Supplementary Note 3. In the end, we ended up filling the unobserved cells with the average of all measurements per channel which results in a smoother input and helps to propagate the information over the full domain.

A scaled version of the mean squared error (MSE) loss is applied to train the model balancing the loss *L*( ⋅ ) between the samples and channels:

1 $$L\left(X,Y,N\right)=\frac{1}{N}{\sum}_{c}{\left(\frac{{X}_{c}-{Y}_{c}}{{\hat{Y}}_{c}}\right)}^{2},$$

where *X* is the network prediction, *Y* the label flow, ${\hat{Y}}_{c}$ the label average per channel of the non-terrain cells, and *N* the number of non-terrain cells. Normalizing the error by the average label value balances the loss for flows of different magnitudes. Without accounting for the number of terrain cells in the loss, a sample with a high ratio of terrain cells would not contribute much to the overall loss. Thus, scaling according to *N* prevents these cases from being underrepresented in the training.

The model is trained using the Adam optimizer^70^ for 3000 epochs. The initial learning rate of 1.0 × 10^−5^ is quartered every 700 epochs.

### Measurement campaign datasets

Each of the three measurement campaign datasets that we used for evaluation are publicly available but require some preprocessing to enable direct comparison with our wind prediction outputs. We convert the data from the different file formats for each measurement campaign to the same gridded format that we use to store the CFD solutions. Each experiment provides terrain data as well as wind measurements collected using static masts equipped with airflow sensors at various heights. The terrain is discretized by querying the raw data using bilinear interpolation in the center of the respective cell. The location of each measurement is converted into the cell coordinates.

WindSeer predicts the wind using the measurements from one mast. The measurements are filled into the corresponding cell and averaged in case of multiple measurements in one cell. The predictions, which are obtained with trilinear interpolation at the sensing locations, are then compared to the measured wind. CNNs allow for variable input sizes, a trait we exploit to predict at a higher spatial resolution for domains with smaller length scales (see Bolund Hill below) at an increased domain size of 384 × 384 × 192 cells. Since the terrain is represented as a Euclidean distance field, this gives WindSeer a sense of the grid resolution and thus the scale of the flow, enabling us to predict the wind at different scales.

The error bars for Fig. 6 are calculated using error propagation from the standard deviations of each axis if not reported for the magnitude^71^.

#### Bolund hill

The data for the Bolund hill experiment containing time-averaged wind velocities and TKE measurements is publicly available (https://www.bolund.vindenergi.dtu.dk/blind_comparison). As Bolund hill exhibits only a small elevation change of 11 m, the default prediction resolution is not sufficient to account for its near-ground measurement locations. As mentioned above, we exploit the multi-scale property of WindSeer and increase the resolution of the prediction grid thirty-fold resulting in a domain ∼ 211 m × 211 m wide and ∼ 73 m tall, giving a corresponding horizontal resolution of 0.55 m and vertical resolution of 0.38 m.

#### Askervein hill

While a digitized version of the Askervein hill topography is available (https://zenodo.org/record/4095052) the wind and TKE measurements had to be manually extracted from the field report^12^. We selected 13 runs measuring the turbulent wind, where the data from most towers is provided (in certain runs data is not reported for all towers). The measurements are averaged over one- to four-hour intervals with varying flow magnitudes and directions. The domain size of 1584 m × 1584 m wide and 552 m tall results in a four-fold resolution increase.

#### Perdigão

The Perdigão dataset consists of multiple measurement posts of different heights ranging from 10 m up to 100 m across the valley or along the ridges (https://perdigao.fe.up.pt/). We used the five minute averages and tilt corrected measurements that were recorded throughout the measurement campaign and we consider data from six different days in our evaluation. The tower positions were not stored with sufficient precision in the dataset requiring us to manually correct the positions. We extracted the topography of the hills from the World Elevation Terrain layer provided by Esri using ArcGIS. Perdigão required the largest prediction domain size, 3168 m × 3168 m wide and 1104 m tall, showcasing the wind prediction performance at double the original resolution.

### Inference time experiments setup

We ran the inference time experiments on an Orin AGX, a low power, light weight (623 g including the carrier board and heatsink) and small scale (105 mm × 105 mm × 60 mm) single-board computer that can be carried by a small scale sUAV. We set up the Orin AGX with the Jetpack 5.1 software kit that includes CUDA 11.4 and cuDNN 8.6.0 and installed PyTorch 2.0. During the evaluation we ran the Orin in the maximum power mode (60 W) using all 12 CPU cores.

### sUAV flight tests

We used three Multiplex EasyGlider4 airframes equipped with the Pixhawk 4 autopilot^72^ using the high quality ADIS16448 inertial measurement unit (IMU) and the u-blox M9N GNSS module for autonomous navigation. We configured the main height source of our modified PX4 autopilot^73^ to the GPS height and use the barometric pressure as a fallback. An extension to the guidance law adjusting the airspeed ensured safety during strong wind conditions^5^. We used a custom designed pitot tube with the Sensirion SDP31 differential pressure sensor and Hall sensor airflow vanes to enable measuring the 3D wind vector. Refer to Supplementary Note 4 for more details about the airflow sensing setup and calibration procedure. We used a ground station computer with QGroundControl to control and navigate the sUAV. While the default PX4 state estimator could be extended to estimate the 3D wind we opted for an offline flight path reconstruction (FPR) pipeline using an iterated extended Kalman filter (see a similar problem definition in^74^). The offline FPR pipeline allowed us to generate high quality estimates for validating our approach and to adjust the estimation pipeline post flight.

We gathered wind data from flights at three test sites in Switzerland. The first test site at Chasseral is one of the most topographically isolated mountains in Switzerland and is located in the Jura mountains (47^°^ 07’ 38” N, 7^°^ 02’ 47” E, 1548 m above mean sea level (AMSL)). The other test sites are located on the ridges of the Oberalppass (46^°^ 39’ 24” N, 8^°^ 40’ 21” E, 2069 m AMSL) and Gotthardpass (46^°^ 34’ 17” N, 8^°^ 33’ 33” E, 1960 m AMSL) in the Central Swiss Alps. These were chosen to evaluate the prediction performance for domains surrounded by complex terrain. The spatial constraints allowed for two sUAVs to simultaneously collect wind data at Oberalppass and Gotthardpass and three sUAVs at Chasseral. The sUAVs were flown simultaneously in circular loiter patterns with a radius of 100 m leading to lateral separation between the planes of up to 800 m and measurements in different flow regimes. We planned the flights based on NWP forecasts ensuring good flight (no precipitation, fog or clouds) and stable wind conditions (wind magnitude below the cruise speed of 10 ms^−1^, direction and magnitude near-constant over multiple hours).

We use two modes to convert the raw wind estimates to the WindSeer input. In the first mode we generated the input by averaging the measurements over one loiter pattern to generate a single wind estimate. This time-averaged data, similar to the averaged data in the measurement campaigns with static masts, helped to reduce the noise and sensitivity to sensor calibration on the input measurements. The observations from the different sUAVs and multiple loiters enabled us to compute the average prediction error and the correlation for each flight experiment to see if the flow trends are well predicted. In the second mode the input is composed of the wind data from a 120 s window of one sUAV to predict the wind along the flight path within the next 120 s window for both the input (itself) and the validation sUAVs. This sequential time-windowed setup allowed us to qualitatively evaluate the WindSeer performance along the flight paths. A high-resolution elevation map provided by SwissTopo^40^ was used to construct the terrain for the WindSeer input.

### Error metrics

In our experiments, we use a range of metrics to evaluate the performance of WindSeer. Aside from commonly used metrics such as MSE, RMSE, Pearson correlation coefficient (*r*) and bias, in this section we define the less common error metrics used in this work.

In the CFD experiments we compute the relative error *ϵ*_*rel*_ for a property *P* over all non-terrain cells as follows:

2 $${\epsilon }_{rel}=\frac{1}{{N}_{wind}}{\sum}_{{N}_{wind}}\frac{\parallel {P}_{WindSeer}-{P}_{CFD}\parallel }{\parallel {P}_{CFD}\parallel },$$

where *N*_*wind*_ is the number of non-terrain cells, and ∥ ⋅ ∥ indicates *ℓ*^2^-norm. This ensures the metric is not skewed by the number of terrain cells in a sample as there the error is not defined. The properties we report are the TKE and velocity magnitude (*ℓ*^2^-norm of a 3D wind vector (*u*, *v*, *w*)).

The averaged speedup error *ϵ*_*speedup*_ as presented in Bechmann et al.^10^ is defined as the average of the speedup error *R*_*S*_ over all measurements:

3 $${\epsilon }_{speedup}=\frac{1}{N}{\sum}_{N}\parallel {R}_{S}\parallel .$$

The speedup error is the difference of the predicted fractional speedup Δ*S*_*p*_ and the measured fractional speedup Δ*S*_*m*_ for that location:

4 $${R}_{S}=100\left(\Delta {S}_{p}-\Delta {S}_{m}\right).$$

The fractional speedup Δ*S* is computed as the velocity difference at a certain location to a reference speed normalized by the reference speed:

5 $$\Delta S=\frac{{s}_{{z}_{agl}}-{s}_{0,\,{z}_{agl}}}{{s}_{0,\,{z}_{agl}}},$$

where ${s}_{{z}_{agl}}$ is the speed at the mast location and ${s}_{0,{z}_{agl}}$ is the reference speed at the same height above ground *z*_*agl*_. These speeds can either be predictions from WindSeer (in the case of *S*_*p*_) or measurements (*S*_*m*_).

### Supplementary information

Supplementary Information

Peer Review File

Description of Additional Supplementary Files

Supplementary Movie 1

### Source data

Source Data

## Data Availability

The processed CFD data and real campaign and sUAV flight measurements are available at https://projects.asl.ethz.ch/datasets/doku.php?id=nature_2024_windseer and the ETH Research Collection^75^: https://www.research-collection.ethz.ch/handle/20.500.11850/658323. The underlying data for the figures and tables can be either obtained in the Source Data file or step by step instructions in Supplementary Note 5 on how to extract the data are provided. Source data are provided with this paper.

## Code availability

The code is available on github: https://github.com/ethz-asl/WindSeer or Zenodo^76^: 10.5281/zenodo.10844690.
