# Supplementary material for: WindSeer: real-time volumetric wind prediction over complex terrain aboard a small uncrewed aerial vehicle
Source: Nat Commun. 2024 Apr 25;15:3507. doi: 10.1038/s41467-024-47778-4 (PMC11045725; doi:10.1038/s41467-024-47778-4)
Supplement: Supplementary file 1 — Supplementary Information [file 41467_2024_47778_MOESM1_ESM.pdf]

Supplementary Information for WindSeer: Real-time volumetric wind prediction over complex terrain aboard a small uncrewed aerial vehicle

Florian Achermann[1*], Thomas Stastny[1*], Bogdan Danciu[1], Andrey Kolobov[2], Jen Jen Chung[1,3], Roland Siegwart[1] and Nicholas Lawrance[1,4*]

[1]Autonomous Systems Lab, ETH Zurich, LeonhardRämistrasse 2101, Zurich, 8092, Zurich, Switzerland.
[2]Microsoft Research, Microsoft, One Microsoft Way, Redmond, WA-98052, USA.
[3]School of Electrical Engineering and Computer Science, The University of Queensland, Staff House Road, Brisbane, 4072, Queensland, Australia.
[4]Robotic Perception and Autonomy, Data61, CSIRO, 1 Technology Court, Brisbane, 4069, Queensland, Australia.

*Corresponding author(s). E-mail(s): acfloria@ethz.ch; tstastny@ethz.ch; nicholas.lawrance@data61.csiro.au;

Supplementary Note 1: Wind characteristics terminology

The complex wind around terrain has some typical flow regions and we want to establish common terms for some of these regions as shown in Supplementary Figure 1. The side of the terrain where the wind direction points toward the hill is called the *upwind* side since it typically exhibits mostly rising winds. At the highest point of the terrain (*hill top*) the wind speeds up and higher wind magnitudes can be measured. The *lee side* of the terrain/hill describes the region where the wind direction typically points away from the hill top. This region is highly turbulent and can form multiple modes with completely different characteristics. Under certain conditions the flow can follow the terrain downhill resulting in prevailing downwinds. In other conditions a *lee side rotor* can form, where the wind follows a circular motion close to the terrain behind the hill. At a larger scale of multiple kilometers and under very specific conditions, mountain waves with multiple rotors can form [1].

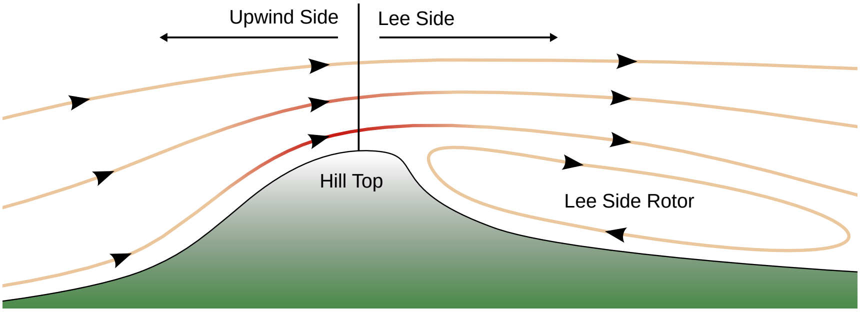

Supplementary Figure 1 Example of wind over a hill resulting in a lee side rotor.

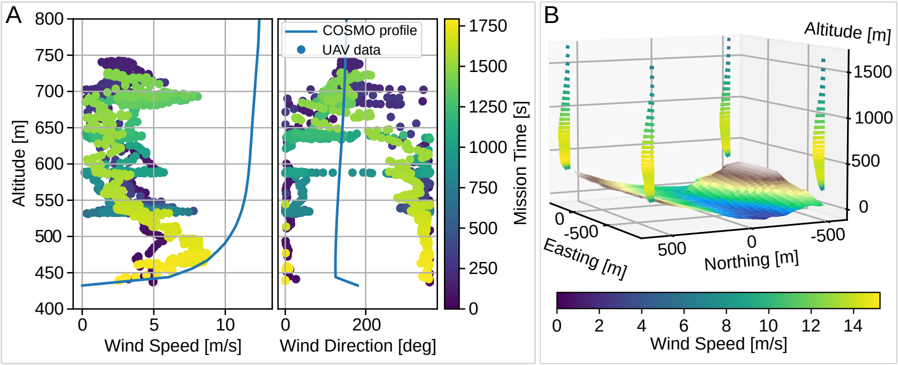

Supplementary Figure 2 (A) small uncrewed aerial vehicle (sUAV) wind measurements compared to the NWP along different flight altitudes showing the mismatch in direction and wind speed. (B) The coarse terrain representation in the NWP causes offsets in the prediction altitudes to the terrain. Source data are provided as a Source Data file.

Supplementary Note 2: NWP data as WindSeer input

Our initial hypothesis was to train WindSeer based on the known high-resolution terrain and predictions from large scale numerical weather prediction (NWP). The Swiss COSMO 1 model provides predictions with a horizontal resolution of 1.1 km [2]. The elevation data used in the NWP models, such as GLOBE [3], is an aggregation of available high-resolution terrain sources (usually the mean or median of the high-resolution data within one cell). This smoothed topography representation neglects smaller scale terrain features and therefore only provides meaningful results at a scale of multiple cells/kilometers.

We conducted a test flight to evaluate how well the NWP of one cell matches the wind measured by an sUAV. We measured the wind at one grid point of the Swiss COSMO-1 model[1] close to Flüelen (46° 53' 33" N, 8° 36' 45" E, 436 m above mean sea level (AMSL)). While Flüelen is located within the Swiss Alps, this particular test site is bordered on one side by a lake and surrounded by flat and smooth terrain within a 1 km radius resulting in a good match between the NWP terrain model and the high-resolution terrain.

As visible in Supplementary Figure 2 A), the COSMO-1 NWP poorly represents the sUAV data for both the magnitude and wind direction. Obviously in different conditions the NWP might fit the measurements better. However, this implies that, depending on the case, the NWP may or may not be accurate. Thus, WindSeer needs another, more reliable source for its wind prediction data. In addition, the coarse representation of the terrain can result in large altitude offsets of the NWP compared to the actual terrain in the presence of large elevation changes, see Supplementary Figure 2 B).

The NWP data may provide supplemental information to WindSeer if used together with the sparse measurements. However, first the mapping between the NWP data to the actual flow needs to be established. This would be a highly data-driven task, and if that connection is too noisy, WindSeer might learn to ignore the NWP input data altogether.

Supplementary Note 3: WindSeer ablation study

We evaluated the effect of varying certain hyperparameters in the training pipeline on the model performance on a test set of previously unobserved computational fluid dynamics (CFD) samples. The baseline model parameters are shown in Supplementary Table 1, note that these parameters are different from the finalized WindSeer version. We used the average error norm over all non-terrain cells averaged over all samples in the test set as our metric to compare the models.

---

[1] https://www.meteoschweiz.admin.ch/home/mess-und-prognosesysteme/warn-und-prognosesysteme/cosmo-prognosesysteme.html

Supplementary Table 1 Baseline hyperparameter set used in the ablation study.

Hyperparameter	Value
learning rate	1.0×10^{-5}
learning rate decay	0.25 every 700th epoch
learning epochs	1500
learning batch size	35
max Gaussian noise std	0 %
max bias magnitude	0 %
trajectory min length	3 cells
trajectory max length	50 cells
model depth	4
pooling method	strided convolution
input no measurement value	mean
input use u_z	true

Models trained with different pooling methods (max-pooling (MP), average-pooling (AP), convolution with strides) perform comparably with a slight edge for the pooling methods over the convolution with strides (1.1 % error reduction). The model using only the horizontal wind measurement (NUZ) outperforms the baseline (BL) model, which uses the vertical measurements as well, by 2.6 %. We also varied the input trajectory lengths up to a length of 500 cells (LT). Networks trained on longer trajectories perform 13.6 % better even if they are evaluated exclusively on short trajectories with lengths of up to 50 cells.

Input noise ablation study

Realistic wind measurements are subject to noise. We model the sensor noise with a zero-mean Gaussian distribution and the sensor miscalibration with a constant bias. We evaluated the robustness of the model to different levels of such noisy input. Doing so we trained multiple models (BL architecture) with varying levels of input noise. We varied the standard deviation of the Gaussian noise between 0 % and 80 % of the average flow magnitude of the respective sample; we varied the bias between 0 % and 50 % of the flow magnitude. We then evaluated the models in two ways: First we evaluated them on the test set with the same noise distribution they observed during training. Since this is not a fair comparison, as predicting with high-noise levels is more difficult than low-noise data, we also evaluated all models against perfect data (no noise added). The results of the experiment are displayed in Supplementary Figure 3 A). In general, higher input noise indicates higher prediction errors, but up to a level of 10 % Gaussian noise and bias we observed similar errors. When evaluating the models on the perfect input we can see that the low-level noise models (up to 10 % bias and 30 % Gaussian noise) perform comparably to the baseline model trained without noise (Supplementary Figure 3 B)). Thus training models with a too high noise level will also negatively impact the performance when they are provided with perfect input data.

CFD prediction results of different WindSeer variants

In our evaluation we considered six variations of WindSeer [ZD4, ZD6, AD4, AD6, VD4, VD6] by varying the fill value and network depth. The fill indicator (Z, A, V) indicates how the wind speed input channels are filled for the cells with no measurements. We tested fill values of: zero (Z), the average of all measurements per channel (A), and the Voronoi tessellation presented in [4] (V) (essentially the nearest measurement value). The network depth indicates the number of pooling/upsampling layers in the encoder/decoder of WindSeer and we evaluated depths of four (D4) and six (D6) resulting in receptive field sizes of 175 and 703 respectively. The models were trained using the Adam optimizer [5] for 3000 epochs except for AD6, where the model after 1000 epochs was chosen as further training showed increasing validation loss, suggesting over-fitting.

We used the same input noise distribution as observed during training (Gaussian noise and random bias). Supplementary Figure 3 C) shows the distribution of the relative velocity magnitude and TKE prediction errors over the full flow domain on the left side (blue) and excluding the lowest four cells above the terrain on the right side (green). These latter

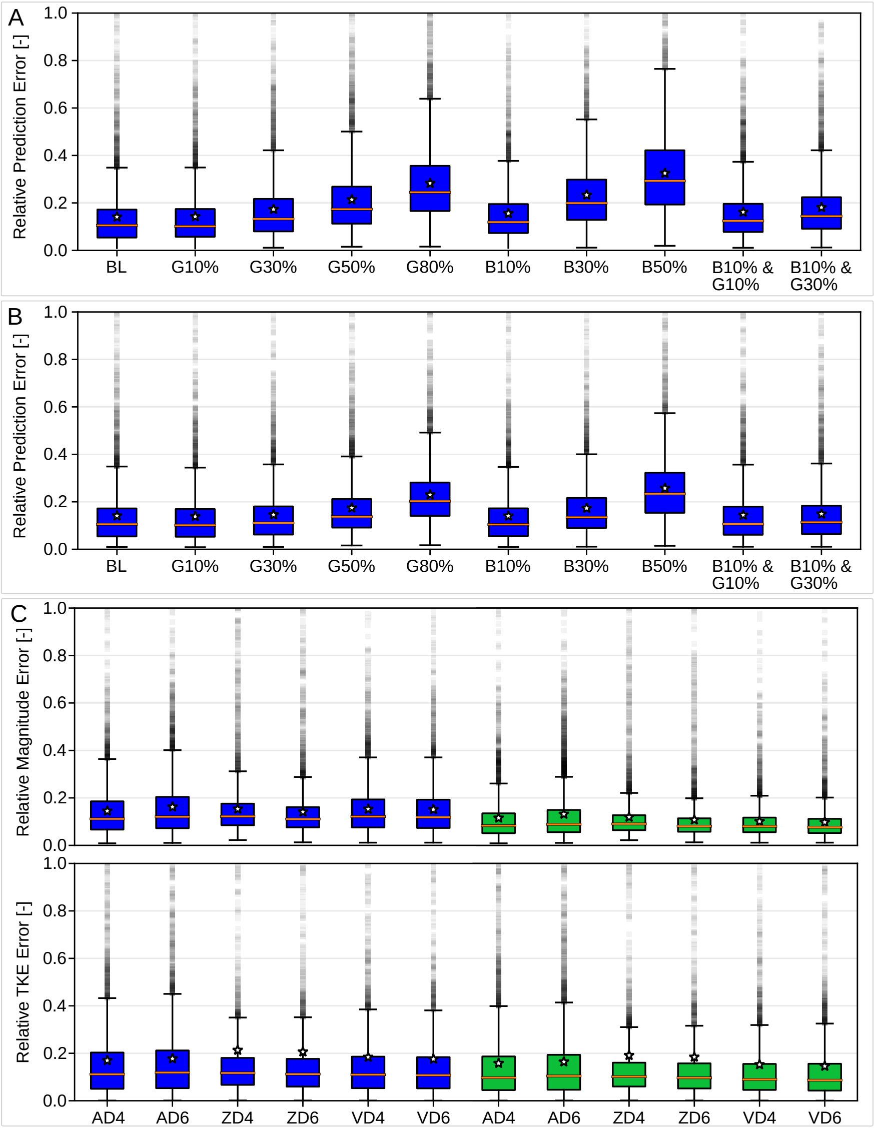

Supplementary Figure 3 Ablation study results on the test set containing 4764 samples. (A) Models trained with different levels of Gaussian noise and biases and evaluated with the same noise distribution used during training. (B) The same models as in A but evaluated without noise on the input data. (C) Wind magnitude and turbulence kinetic energy (TKE) relative prediction errors of the WindSeer variants on the CFD test set on the full domain (left, blue). In contrast to the velocity errors, excluding the closest cells to the terrain does not change the prediction error (right, green) for the TKE. Boxes extend from the first to the third quartiles of data. Median is indicated by a line and mean by a star. Whiskers extend to the extrema data inside 1.5 times the interquartile range beyond the first and third quartiles. Outliers (outside the whiskers) are individually plotted. Source data are provided as a Source Data file.

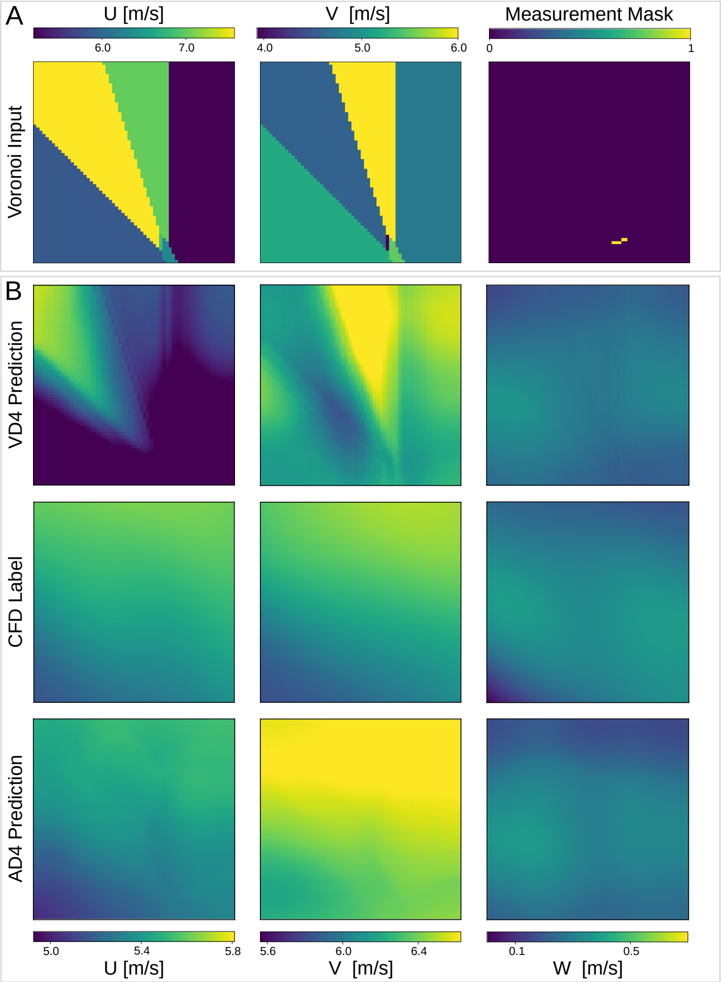

Supplementary Figure 4 (A) A horizontal slice through the domain showing the input to the Voronoi tessellation WindSeer variant (VD4). The measurement mask highlights the cells containing measurements in red (B) the resulting prediction containing strong artifacts along the cell edges especially on the horizontal wind. The CFD label flow and the AD4 prediction. The AD4 prediction still shows some artifacts due to the input measurements albeit with much smaller significance.

results (equivalent to only scoring the network output above an altitude of 46 m) illustrate the predictive performance for realistic sUAV flight regimes. There, all WindSeer variants produced more accurate wind velocity predictions (median error reduction AD4: 11.1 % to 8.3 %, AD6: 12.0 % to 8.9 %, ZD4: 12.2 % to 9.0 %, ZD6: 11.0 % to 8.1 %, VD4: 12.1 % to 8.0 %, VD6: 11.9 % to 7.6 %). In contrast to the velocity errors, the TKE predictions do not significantly change on the reduced prediction volume since the computed TKE values close to the terrain tend to be smoother than the velocity values, thus suffering less from resolution differences between WindSeer and the CFD simulations. All the WindSeer variants result in a similar median between 10.8 % to 11.9 %. Depending on the metric different models perform best. The averaging input models score the lowest mean error while the Voronoi variants yield the lowest median error. The zero-fill variants are most consistent with the lowest 75th percentile.

Overall, as evident in Supplementary Figure 3 C), there is no significant performance difference between the metrics of the WindSeer variants. However, a qualitative assessment of the predicted wind fields reveals that the Voronoi tessellation models (VD4 and VD6) show strong artifacts along the partition borders in certain cases. In contrast, the other WindSeer variants either do not exhibit such artifacts or are effected at a much smaller scale. These artifacts are a result of the noisy measurements being close to each other resulting in large differences between the Voronoi partitions. In Supplementary Figure 4 we show one such case and the resulting predictions for the VD4 and AD4 WindSeer variants. These results indicate that while Voronoi tessellation has been shown to work with sparse input data for flow prediction [4], in our setting with highly noisy measurements from only a small sub-region of the domain, this input modality can result in predictions containing artifacts. Thus, we further evaluate the artifact-free WindSeer variants on the real wind data (AD4, AD6, ZD4, ZD6).

Measurement campaign results of different WindSeer variants

We evaluated the performance of the different WindSeer variants on the data from the Bolund, Askervein, and Perdigão data and report the prediction error and correlation averaged for certain wind cases, as in Tab. 4. The changes in the prediction grid as outlined in Section "Measurement campaign datasets" resulted in higher sparsity levels in the range of 3.5×10^{-6} % to 3.2×10^{-5} % compared to the training density of 1.1×10^{-3} % to 0.19 %. The model variants using average-filling (AD4, AD6) could generalize to this much sparser input data in contrast to the zero-fill models (ZD4, ZD6), which severely underpredicted the wind regardless of the measurement values. Thus, we only compared the two performant WindSeer variants against an averaging baseline (AVG) that assumes the wind and TKE are constant and predicts the average of all measurements over the full domain and report the prediction errors and correlations in Tab. 4. The AD4 variant resulted in better wind magnitude predictions, while the AD6 predicted the vertical wind better. The TKE is predicted with a lower error with the AD6 variant but also with lower correlation values compared to AD4. Overall, in most cases both WindSeer variants performed similarly to each other, explaining the small difference in the averaged error over all cases for the three metrics. We chose the AD4 variant as our WindSeer model as it did not show the over-fitting during training.

		Error S $[m/s]$			Error W $[m/s]$			Error TKE $[m^2/s^2]$		
Terrain	Case	AVG	AD4	AD6	AVG	AD4	AD6	AVG	AD4	AD6
Bolund	90	1.80	**1.58**	1.61	0.85	**0.58**	0.62	1.91	1.33	**1.19**
	239	2.80	2.50	**2.49**	0.66	**0.34**	0.37	2.67	1.68	**1.66**
	255	3.24	**2.47**	2.61	0.85	**0.44**	0.46	3.43	2.12	**2.06**
	270	3.77	**2.79**	2.94	0.95	0.51	**0.48**	5.14	3.43	**3.35**
Askervein	TU25	2.58	**2.39**	2.44	1.10	0.37	**0.31**	0.61	0.41	**0.34**
	TU30A	1.14	**0.98**	1.20	0.41	0.26	**0.25**	1.38	0.72	**0.54**
	TU30B	1.80	**1.46**	2.13	0.51	0.41	**0.39**	2.82	1.40	**1.18**
	TU01A	3.26	**2.83**	3.21	1.41	0.52	**0.46**	1.89	1.06	**0.96**
	TU01B	3.24	**2.74**	3.12	1.37	0.48	**0.42**	1.64	0.98	**0.87**
	TU01C	3.55	**3.08**	3.42	1.23	0.45	**0.41**	1.17	0.72	**0.68**
	TU01D	4.21	**3.71**	4.04	1.26	0.47	**0.42**	1.62	1.17	**0.99**
	TU03A	5.29	**4.70**	5.00	1.74	0.64	**0.55**	2.04	1.31	**1.15**
	TU03B	4.90	**4.41**	4.70	1.54	0.54	**0.46**	1.82	1.21	**1.04**
	TU05A	1.91	**1.89**	1.92	0.76	0.31	**0.27**	1.73	0.99	**0.76**
	TU05B	1.18	**1.00**	1.12	0.31	**0.26**	0.28	1.40	0.63	**0.51**
	TU05C	0.93	**0.93**	1.01	0.34	0.25	**0.25**	1.09	0.43	**0.39**
	TU07B	3.42	3.27	**3.18**	1.59	0.49	**0.41**	2.44	1.85	**1.43**
Perdigão 2017-05-09	13:30-13:35	2.91	2.27	**2.17**	0.85	**0.57**	0.58	-	-	-
	17:10:17:15	4.41	3.33	**3.12**	1.22	0.88	**0.86**	-	-	-
	17:00-18:00	3.06	2.37	**2.24**	0.80	**0.56**	0.58	-	-	-
Perdigão 2017-05-12	01:00-02:00	2.82	2.31	**2.23**	0.52	0.42	**0.40**	-	-	
	17:00-18:00	2.76	2.23	**2.11**	0.70	0.57	**0.47**	-	-	-
	19:45-19:50	1.15	**0.90**	0.91	0.21	**0.16**	0.18	-	-	-
Perdigão 2017-05-16	07:00-08:00	1.58	1.16	**1.15**	0.27	0.27	**0.22**	-	-	
	11:40-11:45	0.85	0.77	**0.76**	0.33	0.27	**0.27**	-	-	-
	12:40-12:45	0.86	0.75	**0.74**	0.29	0.22	**0.22**	-	-	-
	20:00-21:00	1.35	**0.97**	1.04	**0.22**	0.31	0.29	-	-	-
Perdigão 2017-05-18	14:35-14:40	2.14	1.82	**1.75**	0.41	0.36	**0.33**	-	-	-
	20:00-21:00	1.54	**1.08**	1.11	**0.17**	0.19	0.20	-	-	-
	22:00-23:00	1.52	**1.13**	1.18	0.17	0.17	**0.16**	-	-	-
Perdigão 2017-05-20	03:15-03:20	3.59	**2.63**	2.77	**0.41**	0.55	0.46	-	-	-
	10:00-11:00	2.20	1.84	**1.75**	0.51	0.42	**0.38**	-	-	-
	12:20-12:25	1.93	1.71	**1.61**	0.58	0.43	**0.41**	-	-	-
Perdigão 2017-06-08	00:00-01:00	2.68	**1.87**	2.03	0.35	0.38	**0.33**	-	-	-
	12:40-12:45	1.20	0.90	**0.87**	0.31	**0.22**	0.24	-	-	-
	14:00-15:00	2.49	1.77	**1.64**	0.74	**0.42**	0.43	-	-	-
Total average		2.50	**2.07**	2.13	0.72	0.41	**0.38**	2.05	1.26	**1.12**

		Correlation S			Correlation W			Correlation TKE		
Terrain	Case	AVG	AD4	AD6	AVG	AD4	AD6	AVG	AD4	AD6
Bolund	90	-	**0.72**	0.67	-	0.50	**0.64**	-	0.58	**0.60**
	239	-	0.68	**0.73**	-	**0.76**	0.75	-	0.86	**0.93**
	255	-	0.82	**0.85**	-	0.72	**0.76**	-	0.82	**0.89**
	270	-	0.85	**0.91**	-	0.78	**0.84**	-	0.73	**0.78**
Askervein	TU25	-	**0.65**	0.60	-	0.90	**0.96**	-	**0.89**	0.88
	TU30A	-	0.62	**0.68**	-	0.58	**0.74**	-	0.42	**0.54**
	TU30B	-	**0.73**	0.71	-	**0.64**	0.63	-	0.23	**0.50**
	TU01A	-	**0.77**	0.52	-	0.91	**0.92**	-	**0.85**	0.46
	TU01B	-	**0.79**	0.53	-	0.92	**0.93**	-	**0.87**	0.53
	TU01C	-	**0.78**	0.46	-	0.92	**0.93**	-	**0.90**	0.64
	TU01D	-	**0.79**	0.54	-	0.93	**0.94**	-	**0.93**	0.81
	TU03A	-	**0.78**	0.65	-	0.93	**0.95**	-	**0.98**	0.94
	TU03B	-	**0.77**	0.61	-	0.92	**0.95**	-	**0.90**	0.89
	TU05A	-	**0.62**	0.53	-	0.89	**0.91**	-	**0.40**	0.31
	TU05B	-	**0.79**	0.69	-	0.48	**0.53**	-	**0.04**	-0.09
	TU05C	-	**0.66**	0.53	-	0.58	**0.60**	-	**0.14**	-0.05
	TU07B	-	**0.70**	0.66	-	0.90	**0.97**	-	0.40	**0.41**
Perdigão 2017-05-09	13:32:30	-	**0.82**	0.82	-	0.53	**0.57**	-	-	-
	17:12:30	-	0.48	**0.80**	-	0.35	**0.48**	-	-	-
	17:00-18:00	-	0.77	**0.77**	-	0.50	**0.54**	-	-	-
Perdigão 2017-05-12	01:00-02:00	-	**0.76**	0.74	-	0.45	**0.51**	-	-	-
	17:00-18:00	-	**0.81**	0.81	-	0.57	**0.61**	-	-	-
	19:45-19:50	-	0.71	**0.72**	-	**0.57**	0.53	-	-	-
Perdigão 2017-05-16	07:00-08:00	-	0.65	**0.68**	-	**0.67**	0.63	-	-	-
	11:40-11:45	-	0.51	**0.55**	-	**0.22**	0.18	-	-	-
	12:40-12:45	-	**0.48**	0.45	-	0.30	**0.31**	-	-	-
	20:00-21:00	-	**0.68**	0.64	-	0.21	**0.25**	-	-	-
Perdigão 2017-05-18	14:35-14:40	-	0.70	**0.72**	-	0.33	**0.39**	-	-	-
	20:00-21:00	-	**0.84**	0.80	-	0.12	**0.19**	-	-	-
	22:00-23:00	-	**0.74**	0.69	-	0.42	**0.50**	-	-	-
Perdigão 2017-05-20	03:15-03:20	-	0.61	**0.64**	-	**0.30**	0.30	-	-	-
	10:00-11:00	-	0.71	**0.76**	-	**0.46**	0.44	-	-	-
	12:20-12:25	-	0.66	**0.71**	-	**0.45**	0.42	-	-	-
Perdigão 2017-06-08	00:00-01:00	-	**0.69**	0.67	-	**0.44**	0.42	-	-	-
	12:40-12:45	-	0.77	**0.77**	-	0.46	**0.46**	-	-	-
	14:00-15:00	-	**0.82**	0.82	-	0.58	**0.62**	-	-	-
Total average		-	**0.71**	0.68	-	0.59	**0.62**	-	**0.64**	0.59

Supplementary Table 2 Measurement campaigns error results: Absolute prediction errors and correlations for the velocity magnitude (S), vertical wind component (W), and turbulence kinetic energy (TKE) on the measurement campaign datasets of the AD4 and AD6 models compared to the averaging baseline (AVG). The best performing model for each case is highlighted bold.

Flight	Model	Mean Absolute Error W_{hor} [m/s]	Ψ_{hor} [deg]	W_z [m/s]	Correlation W_{hor}	Ψ_{hor}	W_z
Chasseral 1	AVG	0.62	**7.22**	0.53	-	-	-
	AD4	0.77	7.84	0.66	0.54	**-0.09**	0.94
	AD6	**0.61**	7.49	0.55	**0.77**	-0.17	**0.95**
	ZD4	0.87	9.18	0.41	0.26	-0.13	0.95
	ZD6	0.84	9.19	**0.38**	0.33	-0.37	0.95
Chasseral 2	AVG	0.57	**13.6**	0.50	-	-	-
	AD4	0.66	14.7	0.48	**0.50**	**-0.35**	**0.90**
	AD6	**0.48**	14.1	0.47	0.48	-0.46	0.87
	ZD4	0.73	17.2	0.35	0.49	-0.42	0.78
	ZD6	0.74	16.8	**0.31**	0.49	-0.60	0.80
Chasseral 3	AVG	0.55	**9.1**	0.49	-	-	-
	AD4	0.56	9.8	0.39	0.43	**-0.21**	0.82
	AD6	**0.55**	9.5	0.40	**0.51**	-0.33	0.84
	ZD4	0.67	14.0	0.32	0.32	-0.43	0.80
	ZD6	0.65	12.4	**0.30**	0.37	-0.38	**0.84**
Oberalppass	AVG	**0.55**	7.0	0.55	-	-	-
	AD4	1.05	19.7	0.30	0.28	-0.86	**0.81**
	AD6	3.12	7.5	0.35	**0.43**	**0.85**	0.76
	ZD4	0.65	**5.8**	**0.33**	-0.24	0.62	0.78
	ZD6	0.77	7.9	0.34	-0.46	-0.91	0.77
Gotthardpass	AVG	**1.00**	**7.5**	0.21	-	-	-
	AD4	2.55	64.3	0.57	0.26	0.75	0.21
	AD6	1.41	58.3	1.06	**0.71**	**0.98**	0.48
	ZD4	1.40	7.9	0.20	-0.06	0.97	0.48
	ZD6	1.19	7.8	**0.17**	0.33	0.63	**0.88**
All Chasseral Flights	AVG	0.58	**9.98**	0.51	-	-	-
	AD4	0.66	10.78	0.51	0.49	-0.22	**0.89**
	AD6	**0.55**	10.36	0.47	**0.55**	-0.32	**0.89**
	ZD4	0.76	13.46	0.36	0.36	-0.33	0.84
	ZD6	0.74	10.60	**0.34**	0.19	-0.11	0.86
All Flights	AVG	**0.66**	**8.88**	0.46	-	-	-
	AD4	1.12	23.27	0.48	0.40	-0.15	0.74
	AD6	1.23	19.38	0.57	**0.58**	**0.17**	0.78
	ZD4	0.86	10.82	0.32	0.15	0.12	0.76
	ZD6	0.84	9.5	**0.30**	0.09	-0.13	**0.85**

Supplementary Table 3 sUAV flight results: The mean absolute error and the correlation for the horizontal wind magnitude W_{hor}, wind direction Ψ_{hor}, and vertical wind W_z on the loiter-averaged data. The results are the average over all loiters for all planes for the respective flight. The best performing model for each case is highlighted bold.

sUAV results of different WindSeer variants

We present the error metrics for all flights and the different model variants in Tab. 3. All models consistently struggle at predicting the horizontal wind while the vertical wind prediction is much more accurate compared to the baseline. The average-filling models strongly rely on the averaged measurement, thus providing a good representation of the overall flow state. However, in the flight experiments with complex topography, the measured wind does not have this property, therefore the zero-fill models (ZD4, ZD6), that learnt to better encode the measurement locations, outperform the average-filling (AD4, AD6) variants. In this set of experiment we see a slight trend that increasing network depth seems to improve the prediction quality.

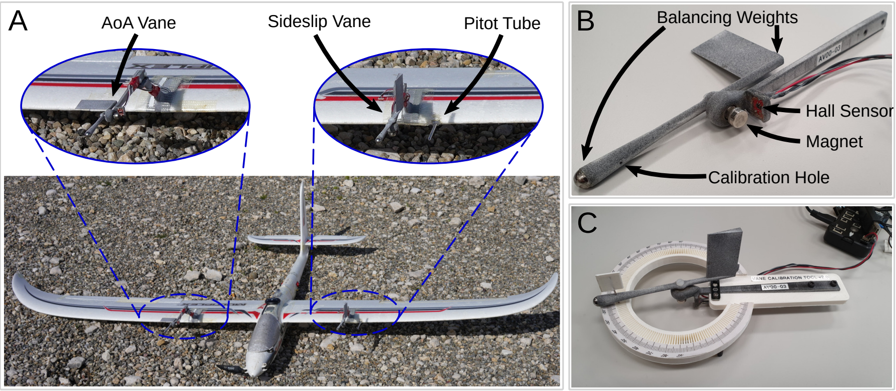

Supplementary Figure 5 (A) The arrangement of the airflow sensors on the sUAV. (B) Components of the airflow vanes. (C) Calibration tool used to determine the mapping from the magnetic flux density to the angle.

Supplementary Note 4: sUAV airflow sensing

In this section we outline the design the airflow vanes used to estimate the full 3D wind aboard the sUAVs. We explain and evaluate the calibration procedure to account for the constant mounting offset and the dynamic aerodynamic biases.

Design and calibration of sUAV airflow vanes

Two custom-designed wind vanes together with a pitot tube measure the 3D airflow. One vane measures the angle of attack (AoA) and the other one the angle of sideslip (AoS) of the airspeed vector relative to the sUAV body reference frame. During flight the wings flex due to maneuvers or wind gusts causing measurement error on the vanes that are larger if the vanes are mounted further towards the wingtips. However, the prop wash makes any placement too close to the fuselage invalid since the vanes need to measure the undisturbed free flow. Therefore we place the vanes approximately one quarter of the wing length away from the fuselage (Supplementary Figure 5 A)).

The vane is 3D printed and balanced using metal weights at the front and back (Supplementary Figure 5 B)). Small ball bearings at the connection axis ensure little friction in the setup and fast response time to changing wind. A diametric radial magnet is mounted at the end of the connection axis resulting in a changing magnetic field (for different angles) that the Hall sensor measures.

The calibration tool, shown in Supplementary Figure 5 C), allowed us to accurately set the vanes to angles with 2° increments, thus gathering accurate data to determine the mapping from the magnetic flux density B. We calibrated each sensor for angles ranging from $-24°$ to $24°$ using a third-order polynomial function. Supplementary Figure 6 shows the measurements and the resulting fit for one wind vane.

sUAV airflow sensing calibration

Raw AoA and AoS measurements are subject to mounting errors as well as aerodynamic effects from the fuselage and the wing. We defined calibration functions based on wind tunnel data provided by Heinrich et al. [6] to estimate the true airflow angles based on the sensor measurements $(\alpha_{raw}, \beta_{raw})$ in the relevant range (AoA between $0°$ to $15°$, AoS between $-10°$ to $10°$):

$$\alpha_{off} = p_{\alpha,0} + p_{\alpha,1} \cdot \alpha_{raw} + p_{\alpha,2} \left(\alpha_{raw} + p_{\alpha,3}\right) \left(v_{Aspd} + p_{\alpha,4}\right), \tag{1}$$

$$\begin{aligned} \beta_{off} = p_{\beta,0} + p_{\beta,1} \left(v_{Aspd} + p_{\beta,2}\right) \left(1 + \tanh\left(p_{\beta,3}\left(\alpha_{raw} + p_{\beta,4}\right)\right)\right) + \\ p_{\beta,5} \cdot \beta_{raw} + p_{\beta,6} \cdot \tanh\left(p_{\beta,7}\left(\Phi + p_{\beta,8}\right)\right), \end{aligned} \tag{2}$$

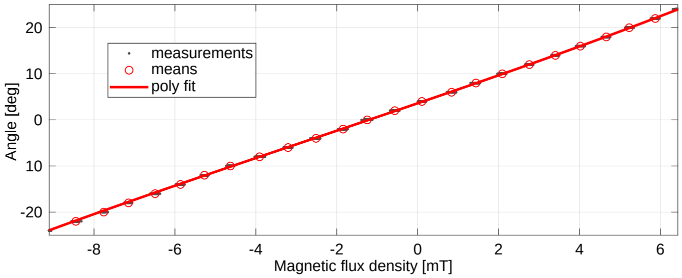

Supplementary Figure 6 Magnetic flux density to angle mapping for one wind vane with the measured data during the calibration procedure. Source data are provided as a Source Data file.

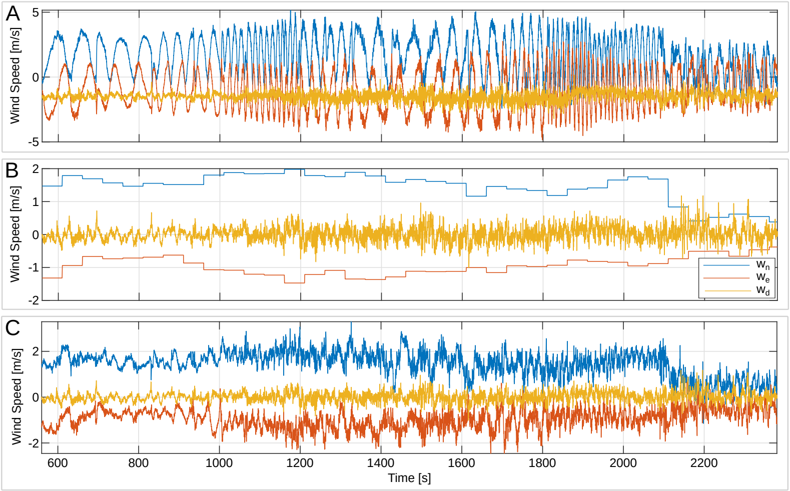

Supplementary Figure 7 (A) The wind estimates based on the raw uncalibrated airflow sensor data. (B) The piecewise horizontal wind and zero-mean vertical wind as optimized during the calibration procedure. (C) The wind estimates after calibrating the airflow sensors. Source data are provided as a Source Data file.

where the p variables are free parameters. For the wind tunnel validation, the parameters were estimated by minimizing the mean squared error (MSE) between the sensor measurements and ground truth airflow angles (orientation of the aircraft using a tunnel-mounted sting, assumed to have very low angular position error). Fitting the wind tunnel data, the base functions result in an MSE for the AoA of 0.45° and 0.83° for the AoS.

However, due to variations in mounts and aircraft, this calibration could not be performed for every sensor installation. Thus, we further defined a calibration routine to estimate the parameters of Eq. 1, 2 based on data gathered during a calibration flight, removing the need to calibrate every sUAV with wind tunnel data. The underlying assumptions that ensure the parameters are observable are that the horizontal wind is piecewise constant and that there is no vertical wind during the calibration flight (calibration flights were performed in as calm flight conditions as possible, usually early morning). We also assume the estimated attitude and global position/velocity are accurate. To cover the different flight regimes our calibration flight consisted of counter-clockwise and clockwise loiter circles of different radii ranging from 30 m to 100 m flown at different airspeeds ($10\,\mathrm{m\,s^{-1}}$ to $16\,\mathrm{m\,s^{-1}}$). We then solved for the calibration parameters and the wind (W_x, W_y) by minimizing the error using

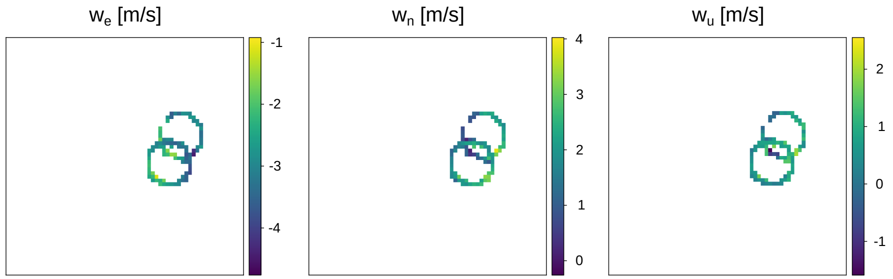

Supplementary Figure 8 A top down view of the binned wind measurements for one sUAV for the first Chasseral flight for two loiters flown at the same altitude.

a nonlinear least-squares solver in the wind triangle over the full flight:

$$\boldsymbol{e} = R\left(\Phi, \Theta, \Psi\right) \begin{bmatrix} v_{Aspd} \\ v_{Aspd} \cdot \tanh\left(\beta - \beta_{off}\right) + l_{x,\beta} \cdot \omega_z - l_{z,\beta} \cdot \omega_x \\ v_{Aspd} \cdot \tanh\left(\alpha - \alpha_{off}\right) - l_{x,\alpha} \cdot \omega_y + l_{y,\alpha} \cdot \omega_x \end{bmatrix} + \begin{bmatrix} W_x \\ W_y \\ 0 \end{bmatrix} - \mathbf{v_{Gnd}}, \quad (3)$$

where $R\left(\Theta, \Phi, \Psi\right)$ is the rotation matrix based on the current attitude, $\mathbf{v_{Gnd}}$ the estimated ground speed vector, and $\omega_{(.)}$ the rotational speed around the respective axis. The offset from the vanes to the autopilot origin is denoted by $l_{x,\beta}$, $l_{z,\beta}$, $l_{x,\alpha}$, and $l_{y,\alpha}$.

Using the uncalibrated measurements from the airflow sensors results in strong oscillations of the estimated horizontal wind (strongly correlated to the loiter frequency) and a vertical estimate with a non-zero mean as visible in Supplementary Figure 7 A) as the sensors are located within the disturbed flow from the wing and airframe. The piecewise linear horizontal and zero-mean vertical wind fit as a result of the airflow calibration pipeline are shown Supplementary Figure 7 B). Although there is no constraint on the difference between the segments in the horizontal wind, the changes are relatively small. This stable, near-constant wind (magnitude and direction) reflects the forecast and observations made from the ground during the flight. The calibration reduces the estimated oscillations in the wind significantly and results in accurately measuring the zero-mean vertical wind (Supplementary Figure 7 C)). However, some correlation between the wind estimates and the loiter frequency remain, indicating that the calibration function could still be improved.

Calibration quality

In contrast to the calibration flights, in the actual data collection flights, the wind estimates from the sUAVs again show some oscillations strongly correlating with the loiter patterns in flight. However, for these data collection flights we expect the wind to vary across different locations so this could be correctly observed changes in the wind field. In Supplementary Figure 8 we display the binned wind observations from two loiters patterns flown at the same altitude next to each other. Especially for the horizontal wind measurements w_e and w_n we can see the same pattern repeating for both loiters with an amplitude of about $1\,\mathrm{m\,s^{-1}}$ in each direction. This pattern indicates that we would expect errors in the horizontal measurements of about $\pm 1\,\mathrm{m\,s^{-1}}$, which are comparable to the observed variation of the measurements between the sUAV and within a single flight. For the vertical wind we do not see such repeating patterns, thus we expect a higher quality of these measurements.

The altitude for the calibration flight of 540 m above mean sea level compared to altitudes of the data collection flights (1600 m to 2200 m) results in 10.5 % to 15.1 % lower air densities at the higher altitudes. Previous work has shown that density changes result in changing flow fields [7, 8]. This could, in part, explain the difficulty to accurately calibrating the airflow sensing if they are located within the disturbed flow field of the air-frame. Therefore, for future flights, the sensors should be placed further away from the wings and fuselage to minimize the aerodynamic disturbances on the sensors, and calibration flights performed at the same altitude as test flights.

Supplementary Note 5: Figure Data Accessibility

In this note we provide step by step instructions on recreating certain figures in the main article and supplemential information based on the publicly available datasets (https://projects.asl.ethz.ch/datasets/doku.php?id=nature_2024_windseer, https://www.research-collection.ethz.ch/handle/20.500.11850/658323.

Figure 2 and Figure 3

To recreate these figures the `test_resampled.hdf5` file, the WindSeer source code, and the `AD4` model folder are required. Then the individual plots for the different cases are generated by executing the following command:

Case 1:

```
python predict.py -ds PATH_TO/test_resampled.hdf5 -model PATH_TO/AD4/ \
    --mayavi -i 1027 -s 68943 --azimuth 210 --elevation 75 --distance 200 \
    --focalpoint 32 32 16 --density --plottools
```

Case 2:

```
python predict.py -ds PATH_TO/test_resampled.hdf5 -model PATH_TO/AD4/ \
    --mayavi -i 610 -s 3 --azimuth 210 --elevation 75 --distance 200 \
    --focalpoint 32 32 16 --density --plottools
```

Case 3a:

```
python predict.py -ds PATH_TO/test_resampled.hdf5 -model PATH_TO/AD4/ \
    --mayavi -i 440 -s 363 --azimuth 210 --elevation 75 --distance 200 \
    --focalpoint 32 32 16 --density --plottools
```

Case 3b:

```
python predict.py -ds PATH_TO/test_resampled.hdf5 -model PATH_TO/AD4/ \
    --mayavi -i 440 -s 65233 --azimuth 210 --elevation 75 --distance 200 \
    --focalpoint 32 32 16 --density --plottools
```

Figure 5 and 6

The Askervein, Bolund, and Perdigão datasets are required as well as the WindSeer source code, and the different WindSeer model folders to generate the data for these figures. The data for the prediction lines can be generated and stored by executing the following command:

```
python3 predict_measurement_campaigns.py -d PATH_TO_DATASET \
    -model_dir PATH_TO_MODEL -model_version latest -i IDX \
    -m TWR --profile --save -e CASE
```

where `PATH_TO_DATASET` is the path to the corresponding dataset file, `PATH_TO_MODEL` is the path to the model and `TWR` and `CASE` specify the tower and case to predict. The `IDX` must be set to 7 for the Askervein experiments, to 10 for Bolund and 6 for Perdigão respectively. To show the predictions for the averaging baseline add `--baseline` and to compute the data for the scatter plots add the `--benchmark` flag. The data for subfigure 6 D is obtained by executing the above command for every 5 minute and 1 hour averaged data case.

Figure 7

To recreate these figures the `UAV_flight_data.zip` file (unzipped), the WindSeer source code, and the `AD4` and `ZD6` model folders are required. In addition you will need to obtain a geotiff of the Chasseral and Oberalppass region. The subfigures can be generated accordingly:

A and C:

First modify the `example_sparse.yaml` file:

```
log:
    filename: 'PATH_TO/EZG3_20210928_Chasseral_flight01_fpr_wind.hdf5'
    geotiff_file: 'PATH_TO/chasseral.tif'
...
    filter_window_size: 1001
...

evaluation:
    mode: 4
...
    validation_file: ['PATH_TO/EZG6_20210928_Chasseral_flight01_fpr_wind.hdf5',
                      'PATH_TO/EZG5_20210928_Chasseral_flight01_fpr_wind.hdf5',]
```

Then execute the following commands:

```
python predict_sparse.py PATH_TO/example_sparse.yaml -model_dir PATH_TO/AD4/ \
    -model_version latest --mayavi

python evaluate_flight_data.py PATH_TO/example_sparse.yaml -model_dir PATH_TO/AD4/ \
    -model_version latest --paths
```

B:

First modify the `example_sparse.yaml` file:

```
log:
    filename: 'PATH_TO/EZG3_20210928_Chasseral_flight01_fpr_wind.hdf5'
    geotiff_file: 'PATH_TO/chasseral.tif'
```

Then execute the following commands:

```
python predict_sparse.py PATH_TO/example_sparse.yaml -model_dir PATH_TO/AD4/ \
    -model_version latest --mayavi

python predict_sparse.py PATH_TO/example_sparse.yaml -model_dir PATH_TO/ZD6/ \
    -model_version e1000 --mayavi
```

D and F:

First modify the `example_sparse.yaml` file:

```
log:
    filename: 'PATH_TO/EZG3_20211011_Oberalppass_flight02_fpr_wind.hdf5'
    geotiff_file: 'PATH_TO/oberalppass.tif'
...
    filter_window_size: 1001
...

evaluation:
    mode: 4
...
    validation_file: ['PATH_TO/EZG5_20211011_Oberalppass_flight02_fpr_wind.hdf5']
```

Then execute the following commands:

```
python predict_sparse.py PATH_TO/example_sparse.yaml -model_dir PATH_TO/AD4/ \
    -model_version latest --mayavi

python evaluate_flight_data.py PATH_TO/example_sparse.yaml -model_dir PATH_TO/AD4/ \
    -model_version latest --paths
```

E:

First modify the `example_sparse.yaml` file:

```
log:
    filename: 'PATH_TO/EZG3_20211011_Oberalppass_flight02_fpr_wind.hdf5'
    geotiff_file: 'PATH_TO/oberalppass.tif'
```

Then execute the following commands:

```
python predict_sparse.py PATH_TO/example_sparse.yaml -model_dir PATH_TO/AD4/ \
    -model_version latest --mayavi

python predict_sparse.py PATH_TO/example_sparse.yaml -model_dir PATH_TO/ZD6/ \
    -model_version e1000 --mayavi
```

Figure 8

To recreate these figures the `UAV_flight_data.zip` file (unzipped), the WindSeer source code, and the `AD4` and `ZD6` model folders are required. In addition you will need to obtain a geotiff of the Chasseral and Gotthardpass region. The subfigures can be generated accordingly:

First modify the `example_sparse.yaml` file:

```
log:
    filename: 'PATH_TO/LOGFILE1'
    geotiff_file: 'PATH_TO/GEOTIIFF'
...
    filter_window_size: 1001
...

evaluation:
    mode: 4
...
    validation_file: [LOGFILE2,]
```

Then execute the following commands:

```
python evaluate_flight_data.py PATH_TO/example_sparse.yaml -model_dir PATH_TO/AD4/ \
    -model_version latest --paths
```

For subfigure A set:

```
LOGFILE1: PATH_TO/EZG3_20210928_Chasseral_flight02_fpr_wind.hdf5
LOGFILE2: PATH_TO/EZG6_20210928_Chasseral_flight02_fpr_wind.hdf5
GEOTIFF: chasseral.tif
```

For subfigure B set:

```
LOGFILE1: PATH_TO/EZG3_20210928_Chasseral_flight03_fpr_wind.hdf5
LOGFILE2: PATH_TO/EZG6_20210928_Chasseral_flight03_fpr_wind.hdf5
GEOTIFF: chasseral.tif
```

For subfigure C set:

```
LOGFILE1: PATH_TO/EZG5_20211011_Gotthardpass_flight01_fpr_wind.hdf5
LOGFILE2: PATH_TO/EZG3_20211011_Gotthardpass_flight01_fpr_wind.hdf5
GEOTIFF: gotthardpass.tif
```

Supplementary Figure 4

To recreate this figure the `test_resampled.hdf5` file, the WindSeer source code, and the `AD4` and `VD4` model folders are required. To get the predictions execute:

```
python predict.py -ds PATH_TO/test_resampled.hdf5 -model PATH_TO_MODEL \
    -model_version latest --plottools -i 30 -s 60
```

Then in the gui select the 16th layer in the x-y direction to display the slice shown in the figure.

Supplementary Figure 8

To recreate this figure the `UAV_flight_data.zip` file (unzipped), the WindSeer source code, and the `AD4` model folder are required. In addition you will need to obtain a geotiff of the Chasseral region. First modify the `example_sparse.yaml` file:

```
log:
    filename: 'PATH_TO/EZG5_20210928_Chasseral_flight01_fpr_wind.hdf5'
    geotiff_file: 'PATH_TO/chasseral.tif'

    alt_offset: 190
```

Then execute the following command:

```
python predict_sparse.py PATH_TO/example_sparse.yaml -model_dir PATH_TO/AD4/ \
    -model_version latest --mayavi
```

In the plotting gui select the x-y slice direction and navigate to the 22nd layer to show the one displayed in the figure.

Table 1 and Supplementary Table 3

To recreate the flight results data for the tables the `UAV_flight_data.zip` file (unzipped), the WindSeer source code, and the `AD4` and `AD6` model folder are required. In addition you will need to obtain a geotiff of the Chasseral, Gotthardpass, and Oberalppass region. First modify the `example_sparse.yaml` file by setting the paths to the logfiles and geotiffs according to the flight:

```
log:
    filename: 'LOGFILE1'
    geotiff_file: 'PATH_TO_TIF'
...
    filter_window_size: 1001
...

evaluation:
...
    validation_file: [LOGFILE2, LOGFILE3]
...
    benchmark: true
```

Then execute the following command for each model and flight:

```
python evaluate_flight_data.py PATH_TO/example_sparse.yaml -model_dir PATH_TO_MODEL \
    -model_version latest --loiter
```

References

[1] Durran DR. In: Ray PS, editor. Mountain Waves. Boston, MA: American Meteorological Society; 1986. p. 472–492.

[2] Voudouri A, Khain P, Carmona I, Avgoustoglou E, Kaufmann P, Grazzini F, et al. Optimization of high resolution COSMO model performance over Switzerland and Northern Italy. Atmospheric Research. 2018;213:70–85. https://doi.org/https://doi.org/10.1016/j.atmosres.2018.05.026.

[3] Hastings DA, Dunbar PK, Elphingstone GM, Bootz M, Murakami H, Maruyama H, et al. The global land one-kilometer base elevation (GLOBE) digital elevation model, version 1.0. National Oceanic and Atmospheric Administration, National Geophysical Data Center. 1999;325:80305–3328.

[4] Fukami K, Maulik R, Ramachandra N, Fukagata K, Taira K. Global field reconstruction from sparse sensors with Voronoi tessellation-assisted deep learning. Nature Machine Intelligence. 2021;3(11):945–951.

[5] Kingma DP, Ba J. Adam: A Method for Stochastic Optimization. In: 3rd International Conference for Learning Representations; 2015. .

[6] Heinrich GA, Vogt S, Lawrance NRJ, Stastny TJ, Siegwart RY. In-Wing Pressure Measurements for Airspeed and Airflow Angle Estimation and High Angle-of-Attack Flight. Journal of Guidance, Control, and Dynamics. 2021;0(0):1–13. https://doi.org/10.2514/1.G006412. https://arxiv.org/abs/https://doi.org/10.2514/1.G006412.

[7] Ejeh C, Afgan I, Shittu R, Sakirudeen A, Anumah P. Investigating the impact of velocity fluctuations and compressibility to aerodynamic efficiency of a fixed-wing aircraft. Results in Physics. 2020;18:103263.

[8] Sayed Ahmed SE, Ibrahiem EZ, Mesalhy OM, Abdelatief MA. Effect of attack and cone angels on air flow characteristics for staggered wing shaped tubes bundle. Heat and Mass Transfer. 2015;51:1001–1016.
