# Supplementary material for: WindSeer: real-time volumetric wind prediction over complex terrain aboard a small uncrewed aerial vehicle
Source: Nat Commun. 2024 Apr 25;15:3507. doi: 10.1038/s41467-024-47778-4 (PMC11045725; doi:10.1038/s41467-024-47778-4)
Supplement: Supplementary file 3 — Description of Additional Supplementary Files [file 41467_2024_47778_MOESM3_ESM.pdf]

Description of Additional Supplementary Files

File Name: Supplementary Movie 1
Description: This movie summarises the motivation and approach of the manuscript, provides additional renderings of the predicted and label wind flows, and shows video from the data collection and corresponding predictions for the sUAV flights.
