# Supplementary material for: WindSeer: real-time volumetric wind prediction over complex terrain aboard a small uncrewed aerial vehicle
Source: Nat Commun. 2024 Apr 25;15:3507. doi: 10.1038/s41467-024-47778-4 (PMC11045725; doi:10.1038/s41467-024-47778-4)
Supplement: Supplementary file 2 — Peer Review File [file 41467_2024_47778_MOESM2_ESM.pdf]

nature portfolio

Peer Review File

WindSeer: Real-time volumetric wind prediction over complex terrain aboard a small uncrewed aerial vehicle

Open Access This file is licensed under a Creative Commons Attribution 4.0 International License, which permits use, sharing, adaptation, distribution and reproduction in any medium or format, as long as you give appropriate credit to the original author(s) and the source, provide a link to the Creative Commons license, and indicate if changes were made. In the cases where the authors are anonymous, such as is the case for the reports of anonymous peer reviewers, author attribution should be to 'Anonymous Referee' followed by a clear attribution to the source work. The images or other third party material in this file are included in the article's Creative Commons license, unless indicated otherwise in a credit line to the material. If material is not included in the article's Creative Commons license and your intended use is not permitted by statutory regulation or exceeds the permitted use, you will need to obtain permission directly from the copyright holder. To view a copy of this license, visit http://creativecommons.org/licenses/by/4.0/.

Editorial Note: This manuscript has been previously reviewed at another journal. This document only contains reviewer comments and rebuttal letters for versions considered at *Nature Communications*.

REVIEWER COMMENTS

Reviewer #2 (Remarks to the Author):

The authors have addressed the issues raised by the reviewers and added necessary benchmarks to demonstrate the effectiveness of their proposed method. Currently, some of the test errors seem large and there is room for improvement. But as commented in the previous review, the experiments (especially the UAV experiment) in this paper are interesting and the idea is promising for future extension. Therefore, I would recommend publication of the current manuscript.

A few additional comments:

Comment 1 from Reviewer #2:

- The newly-added experiment is good for comparison. First, I understand that predicting the wind flow precisely is difficult, so 20%-30% errors are acceptable. On the other hand, as averaging baseline can achieve a relative error of 33%, can we conclude that the investigated wind flow is nearly stable and uniform?

- The definitions of the errors/metrics can be given in Methods. In Page 10 (article file), the authors mention "The average absolute speedup prediction error for WindSeer is 20.3%". The "absolute XXX error" here is a bit misleading, since percentage is used for relative errors.

- In Supplementary Figure C4, please add the locations of the measurements.

Comment 2 from Reviewer #3:

- Data assimilation is not mentioned in the paper. The concept of data assimilation (both variational methods and filtering methods) is similar to the idea of this paper. I suggest the authors to add comments in the introduction or discussion.

Comment 4 from Reviewer #3:

- I can understand how to encode the unstructured data (e.g., point cloud) into a structured grid that is suitable for the Windseer input. The problem is: what should the resolution of the input structured grid be? In the training dataset, the resolution of the input data is 64x64x64. I suspect that the resolution for inference should be the same. Otherwise (input with high resolution), the error will increase.

Comments to Reviewer #2

- **Comment 1:** *The authors have addressed the issues raised by the reviewers and added necessary benchmarks to demonstrate the effectiveness of their proposed method. Currently, some of the test errors seem large and there is room for improvement. But as commented in the previous review, the experiments (especially the UAV experiment) in this paper are interesting and the idea is promising for future extension. Therefore, I would recommend publication of the current manuscript.*

  Response: Thank you very much for taking your time to review the manuscript. We completely agree that there are potential pathways to lowering the prediction errors in the future. We see at least two ways of achieving this. (1) On the software side, one could use a different training dataset, produced by a more complex time-varying NWP solver, or explore alternative network architectures. (2) On the hardware side, improving the UAV's airflow sensing accuracy should improve the quality of inputs to WindSeer, which would also result in evaluation on cleaner data.

- **Comment 2:** *The newly-added experiment is good for comparison. First, I understand that predicting the wind flow precisely is difficult, so 20%-30% errors are acceptable. On the other hand, as averaging baseline can achieve a relative error of 33%, can we conclude that the investigated wind flow is nearly stable and uniform?*

  Response: The investigated wind flow is *temporally* stable as the measurements in the measurement campaigns are averaged over time periods from minutes to hours. However, the investigated flows are not *spatially* uniform, as Fig. 5 shows. The reported speedup errors are the average of the error over all available sonic cup measurements. Please refer to Fig. 3 A for the locations of the different masts. As Fig. 5 A suggests, there is spatial variation in the input measurements, but there is also a relatively consistent component in the measured wind across all locations. Overall, this lowers the observed speedup factors, which leads to the surprisingly good performance of the averaging baseline that you pointed out.

- **Comment 3:** *The definitions of the errors/metrics can be given in Methods. In Page 10 (article file), the authors mention "The average absolute speedup prediction error for WindSeer is 20.3%". The "absolute XXX error" here is a bit misleading, since percentage is used for relative errors.*

  Response: We agree that the average absolute speedup prediction error might be a bit misleading – we initially adopted this term from the Bolund experiment blind study (Bechmann, 2011). We have now adjusted the naming by removing the "absolute" and added a subsection in the methods chapter explaining the definition of the error metrics.

- **Comment 4:** *In Supplementary Figure C4, please add the locations of the measurements.*

  Response: Thanks for this suggestion. We have added the corresponding input mask highlighting the locations of the measurements. We have also modified the figure by choosing a different slice that contains the majority of the input measurements, 5 out of 7. The other measurements are located in the adjacent layers.

- **Comment 5:** *Data assimilation is not mentioned in the paper. The concept of data assimilation (both variational methods and filtering methods) is similar to the idea of this paper. I suggest the authors to add comments in the introduction or discussion.*

  Response: Thank you for this suggestion, assimilation methods are indeed related to our work in that they are attempting to integrate sparse measurements with a prediction model. We have added a paragraph on data assimilation to the introduction of the manuscript (**highlighted in blue**).

- **Comment 6:** *I can understand how to encode the unstructured data (e.g., point cloud) into a structured grid that is suitable for the Windseer input. The problem is: what should the resolution of the input structured grid be? In the training dataset, the resolution of the input data is 64x64x64. I suspect that the resolution for inference should be the same. Otherwise (input with high resolution), the error will increase.*

  Response: Although trained with only one specific resolution and grid size (64x64x64), our experiments show that WindSeer is capable of accurately performing inference at significantly higher input resolutions (Perdigao 2x, Askervein 4x, Perdigao 30x) and larger grid sizes (384x384x192). We can conclude that WindSeer is able to handle different grid parameterizations but the exact parameterization for a certain use case depends on the setting (target domain size, required resolution, available compute, …). We have added a short note in the discussion to highlight these properties of WindSeer.

General Remarks

- In the manuscript we sometimes used "manned/unmanned" and sometimes "crewed/uncrewed". For consistency, we have changed all instances of the former to the latter.

REVIEWERS' COMMENTS

Reviewer #2 (Remarks to the Author):

The authors have addressed the issues raised by the reviewers and made corresponding revisions. I would recommend publication of the current manuscript in NC.

Comments to Reviewer #2

- **Comment 1:** *The authors have addressed the issues raised by the reviewers and made corresponding revisions. I would recommend publication of the current manuscript in NC.*

  Response: Thank you very much for taking your time to review the manuscript.
